# Gut microbiome-mediated primary and acquired resistance to immune checkpoint inhibitors in MSI-H/dMMR colorectal cancer: mechanisms, biomarkers, and therapeutic implications-a narrative review

**DOI:** 10.3389/fcimb.2026.1907957

**Published:** 2026-09-03

**Authors:** Marko Marjanović, Nemanja Maletin, Biljana Kukić, Ivan Nikolić, Nemanja Petrović, Jelena Radić, Aleksandar Đurić, Milica Knezević, Božidar Dejanović, Vanja Ćalić

**Affiliations:** 1 Clinic for Medical Oncology, Oncology Institute of Vojvodina, Sremska Kamenica, Serbia; 2 Medical Faculty, University of Novi Sad, Novi Sad, Serbia; 3 Clinic for Gastroenterology and Hepatology, University Clinical Center of Vojvodina, Novi Sad, Serbia

**Keywords:** primary resistance, acquired resistance, colorectal cancer, gut microbiome, immune checkpoint inhibition, microsatellite instability

## Abstract

**Introduction:**

Immune checkpoint inhibitors (ICIs) have transformed the management of microsatellite instability-high/deficient mismatch repair (MSI-H/dMMR) colorectal cancer (CRC). However, a substantial proportion of patients exhibit primary resistance or eventually develop acquired resistance, highlighting the need for a better understanding of the biological mechanisms influencing therapeutic response. Increasing evidence suggests that the gut microbiome–immune axis is an important regulator of antitumor immunity through complex interactions among microbial communities, microbial metabolites, host immunity, and the tumor microenvironment.

**Main body:**

This narrative review summarizes current evidence regarding the role of the gut microbiome–immune axis in mediating primary and acquired resistance to immune checkpoint inhibition in MSI-H/dMMR CRC. We discuss the physiological interactions that maintain immune homeostasis and review the functional mechanisms through which alterations in microbial metabolic pathways, including short-chain fatty acids, bile acids, tryptophan-derived metabolites, inosine, and polyamines, may influence antitumor immune responses. We further examine microbial composition and functional biomarkers associated with immune checkpoint inhibitor response, together with emerging therapeutic strategies aimed at modulating the gut microbiome–immune axis, including dietary interventions, prebiotics, probiotics, selective antimicrobial approaches, fecal microbiota transplantation, live biotherapeutic products, and next-generation precision microbiome engineering. Finally, we discuss current translational challenges and future research priorities required for successful clinical implementation.

**Conclusion:**

The gut microbiome–immune axis represents a promising area of investigation for understanding resistance to immune checkpoint inhibition in MSI-H/dMMR CRC. While growing evidence supports its biological relevance, much of the current knowledge remains preclinical or is derived from early-phase clinical studies. Future progress will depend on mechanistic investigation, longitudinal multi-omic microbiome profiling, standardized methodologies, prospective biomarker validation, and the rational development of microbiome-directed therapeutic strategies to support precision immuno-oncology.

## 1 Introduction

Immune checkpoint inhibitors (ICIs) have fundamentally transformed the therapeutic landscape of mismatch repair-deficient/microsatellite instability-high (dMMR/MSI-H) colorectal cancer (CRC), producing durable responses and prolonged survival in a molecular subgroup that derives limited long-term benefit from conventional systemic therapy (Boland and Goel, 2010; Le et al., 2017; André et al., 2020; André et al., 2025). Nevertheless, despite the remarkable efficacy of programmed cell death protein-1 (PD-1)-based treatment, up to one-third of patients exhibit primary resistance, while a substantial proportion of initial responders ultimately develop acquired resistance, underscoring the need to identify additional biological determinants of immunotherapy response beyond currently established molecular biomarkers (Le et al., 2017; André et al., 2020; André et al., 2025).

Colorectal cancer remains one of the leading causes of cancer incidence and mortality worldwide, with a steadily increasing burden among younger adults (Murphy and Zaki, 2024; Sung et al., 2026). Although deficient mismatch repair, microsatellite instability, tumor mutational burden, and pathogenic *POLE/POLD1* alterations have substantially improved patient selection for immunotherapy (Le et al., 2017; André et al., 2020; Ambrosini et al., 2024), these biomarkers alone do not fully explain the marked heterogeneity in clinical outcomes observed within the dMMR/MSI-H population (Stemmer et al., 2024). Considerable attention has shifted toward host-related factors capable of modulating antitumor immunity, among which the gut microbiome has emerged as one of the most promising candidates (Routy et al., 2018; Wong and Yu, 2019; Ternes et al., 2020).

The gut microbiome is increasingly recognized as an active regulator of systemic immune homeostasis rather than merely a passive component of gastrointestinal physiology (Belkaid and Harrison, 2017; Iliev et al., 2025). Through continuous interactions among microbial communities, their metabolites, intestinal epithelial cells, innate and adaptive immune populations, and the tumor microenvironment, the gut microbiome–immune axis shapes antigen presentation, T-cell priming, immune cell trafficking, cytokine signaling, and metabolic pathways that collectively influence responsiveness to immune checkpoint inhibition (Belkaid and Harrison, 2017; Wong and Yu, 2019; Tran et al., 2025). Importantly, growing evidence suggests that disruption of this complex network may contribute not only to primary resistance before treatment initiation but also to dynamic mechanisms of acquired resistance that emerge during therapy (Abushukair et al., 2022).

This narrative review critically examines the current evidence supporting gut microbiome-mediated primary and acquired resistance to immune checkpoint inhibitors in dMMR/MSI-H colorectal cancer. Particular emphasis is placed on the gut microbiome–immune axis, spanning microbial taxonomic composition, microbial metabolic function, immune regulatory mechanisms, emerging predictive biomarkers, and microbiome-directed therapeutic strategies. Although the gut microbiome has been investigated across multiple malignancies, this review focuses on MSI-H/dMMR colorectal cancer because it represents the molecular subtype in which immune checkpoint inhibitors have demonstrated the greatest clinical efficacy (Le et al., 2017; André et al., 2020; André et al., 2025) and where microbiome-mediated modulation of antitumor immunity is most likely to have immediate translational relevance. Throughout the review, we distinguish evidence derived directly from dMMR/MSI-H CRC from findings obtained in preclinical CRC models or extrapolated from other solid tumors, while highlighting the major translational challenges and future directions required to integrate microbiome-based approaches into precision immuno-oncology.

## 2 Literature search strategy

This narrative review was conducted through a structured literature search to identify experimental, translational, and clinical studies investigating the relationship between the gut microbiome and immune checkpoint inhibitor response in colorectal cancer, with particular emphasis on mismatch repair-deficient/microsatellite instability-high (dMMR/MSI-H) disease. Electronic searches were performed in PubMed/MEDLINE, Scopus, and Web of Science, supplemented by manual screening of the reference lists of relevant articles.

Searches included combinations of Medical Subject Headings and free-text terms, including “colorectal cancer,” “MSI-H,” “dMMR,” “immune checkpoint inhibitors,” “PD-1,” “PD-L1,” “CTLA-4,” “gut microbiome,” “gut microbiota,” “fecal microbiota transplantation,” “microbial metabolites,” “short-chain fatty acids,” “bile acids,” “inosine,” “tryptophan metabolism,” “polyamines,” “biomarkers,” “primary resistance,” “acquired resistance,” and “immunotherapy resistance.” English-language literature published through May 2026 was considered. ClinicalTrials.gov was additionally searched to identify relevant ongoing or completed interventional studies, and trial records were last verified on 21 July 2026.

Priority was given to randomized clinical trials, prospective observational studies, translational investigations, and high-quality preclinical studies directly evaluating microbiome–immune interactions in colorectal cancer. Studies were considered eligible when they addressed microbiome composition or function, microbial metabolites, host–microbiome immune interactions, biomarkers of immune checkpoint inhibitor response, or microbiome-directed therapeutic strategies relevant to colorectal cancer immunotherapy. Conference abstracts were considered only when they reported clinically relevant findings that had not yet been published in full and were clearly identified as preliminary evidence.

Studies were excluded when they lacked direct relevance to cancer immunity or immunotherapy, provided insufficient methodological information, duplicated data reported in a more complete publication, or focused exclusively on microbiome associations without plausible mechanistic or translational relevance to the scope of this review. Because colorectal cancer-specific evidence remains limited for several emerging concepts, selected mechanistic and clinical studies from other solid tumors, particularly melanoma, non-small cell lung cancer, and renal cell carcinoma, were included when they provided biologically relevant insights. Throughout this review, such evidence is explicitly distinguished from CRC-specific findings to minimize overinterpretation and accurately reflect the current state of knowledge.

## 3 The gut microbiome-immune axis in colorectal cancer

### 3.1 The gut microbiome–immune axis

The human gastrointestinal tract harbors one of the most diverse microbial ecosystems in the body, comprising bacteria, archaea, viruses, fungi, and protozoa that collectively possess a genetic and metabolic capacity substantially greater than that of the human host. Although bacterial communities have been studied most extensively, the biological activity of the gut microbiome is determined not only by its taxonomic composition but also by the dynamic interactions among microorganisms, microbial metabolites, the intestinal epithelium, and the host immune system. These interactions constitute the gut microbiome–immune axis, a bidirectional communication network through which microbial signals influence local mucosal immunity, systemic immune responses, and host metabolism (Belkaid and Harrison, 2017; Ternes et al., 2020; Iliev et al., 2025).

The intestinal epithelium represents the principal interface between luminal microorganisms and the mucosal immune system (Odenwald and Turner, 2017; Allam-Ndoul et al., 2020). Continuous recognition of microbial-associated molecular patterns through pattern-recognition receptors, including Toll-like receptors and nucleotide-binding oligomerization domain-like receptors, permits controlled immune activation while maintaining tolerance toward commensal microorganisms (Iliev et al., 2025). This tightly regulated signaling supports epithelial barrier integrity, antigen sampling, antimicrobial peptide production, and balanced cytokine secretion, thereby contributing to intestinal immune homeostasis (Belkaid and Harrison, 2017; Allam-Ndoul et al., 2020).

Communication within the gut microbiome–immune axis extends beyond the direct recognition of microbial structures. Microbial metabolites function as signaling molecules capable of regulating antigen-presenting and innate immune cells, as well as the differentiation, activation, and function of regulatory and effector T-cell populations (Iliev et al., 2025; Tran et al., 2025). Through these mechanisms, the gut microbiome contributes to local mucosal immunity and systemic immune surveillance and may consequently influence the immune landscape of distant tissues, including colorectal tumors (Fidelle et al., 2023; Iliev et al., 2025).

**Figure 1:**
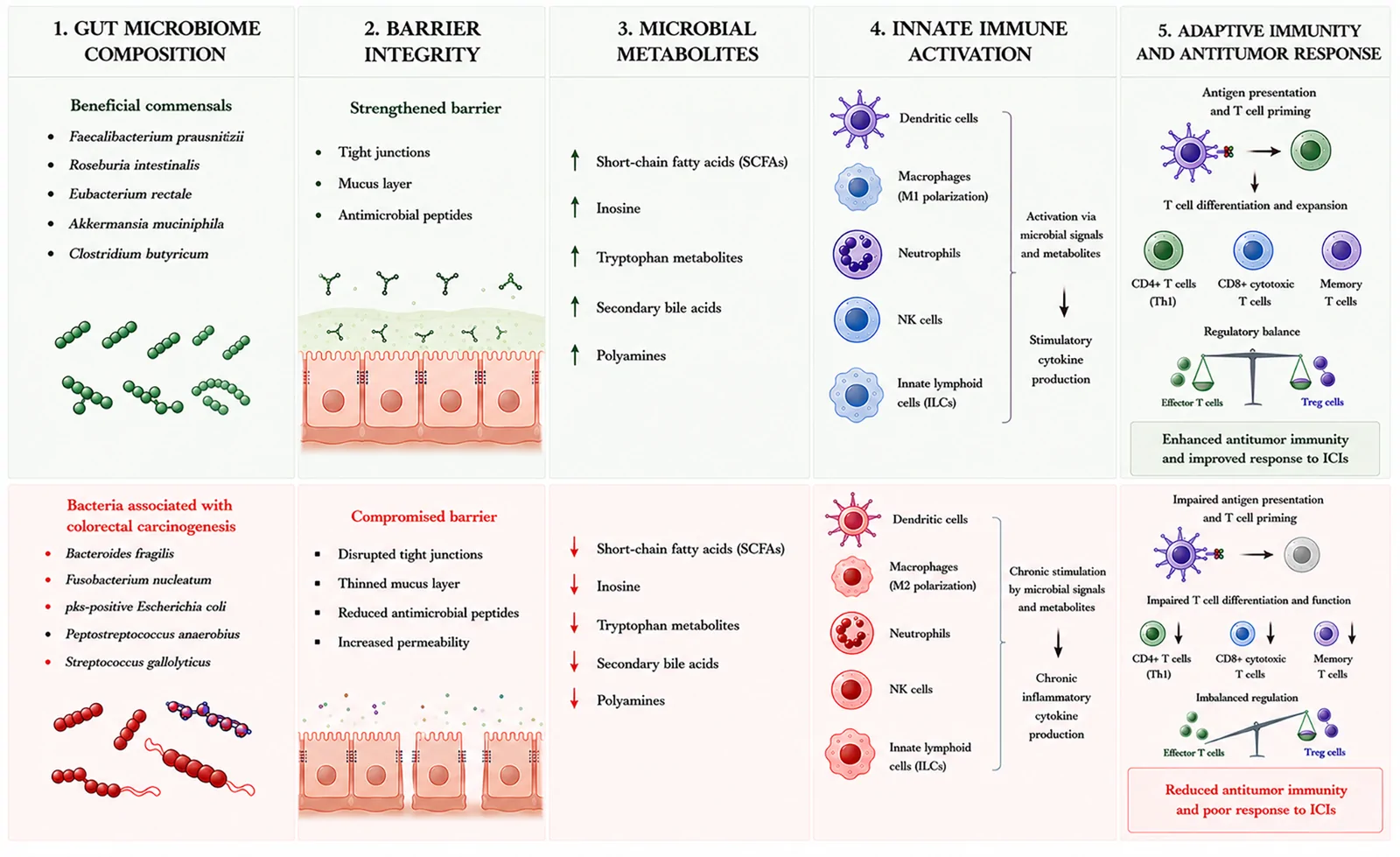
Physiological interactions within the gut microbiome-immune axis.

The coordinated activity of microbial communities, epithelial cells, and innate and adaptive immune populations is therefore important for maintaining effective immune surveillance (Belkaid and Harrison, 2017; Iliev et al., 2025). Disturbances within this network may alter epithelial permeability, immune-cell recruitment, cytokine signaling, antigen presentation, and T-cell function, creating an immunological environment that can facilitate tumor progression (Odenwald and Turner, 2017; Allam-Ndoul et al., 2020; Fidelle et al., 2023) and potentially modify responsiveness to immune checkpoint inhibition (Routy et al., 2018; Almonte et al., 2026). The principal interactions between the gut microbiome, microbial metabolites, epithelial barrier, innate and adaptive immunity, and the tumor microenvironment are summarized in **Figure 1**.

### 3.2 The gut microbiome–immune axis in colorectal carcinogenesis

Colorectal carcinogenesis results from complex interactions among genetic susceptibility, environmental exposures, diet, chronic inflammation, and the gut microbiome (Wong and Yu, 2019; Ternes et al., 2020). Although oncogenic and tumor-suppressor alterations remain central drivers of malignant transformation, accumulating evidence indicates that microbial communities may influence the inflammatory and immune context in which these genetic changes emerge and progress (Arthur et al., 2012).

Several microorganisms have been associated with colorectal carcinogenesis, including enterotoxigenic *Bacteroides fragilis, Fusobacterium nucleatum*, pks-positive *Escherichia coli*, *Peptostreptococcus anaerobius*, and *Streptococcus gallolyticus* (Kostic et al., 2013; Kumar et al., 2017; Chung et al., 2018; Long et al., 2019; Pleguezuelos-Manzano et al., 2020). These organisms do not act through a single shared pathway. Instead, experimental and translational studies suggest that they may contribute through activation of pro-inflammatory signaling, disruption of epithelial-barrier integrity, production of genotoxic compounds, modulation of immune-cell recruitment, and remodeling of the tumor microenvironment. The strength and clinical relevance of these mechanisms vary among individual microorganisms, and many findings remain dependent on preclinical models.

Enterotoxigenic *B. fragilis*, for example, can induce epithelial and inflammatory signaling through the activity of *B. fragilis* toxin, thereby promoting a pro-carcinogenic mucosal environment in experimental models (Chung et al., 2018). *F. nucleatum* has been linked to tumor-promoting inflammation, immune modulation, and treatment resistance, although its abundance and biological effects vary across patient populations and disease stages (Kostic et al., 2013). Certain phylogroup B2 *Escherichia coli* strains harbor the polyketide synthase (*pks*) genomic island, enabling production of the genotoxin colibactin. Colibactin induces DNA double-strand breaks and genomic instability in colonic epithelial cells, thereby promoting colorectal tumorigenesis. More recently, characteristic colibactin-associated mutational signatures have been identified in human colorectal cancers, providing compelling evidence that this bacterial genotoxin contributes directly to colorectal carcinogenesis (Pleguezuelos-Manzano et al., 2020). *Peptostreptococcus anaerobius* has been implicated in colorectal carcinogenesis through activation of pro-inflammatory signaling pathways and modulation of the tumor immune microenvironment, thereby promoting epithelial proliferation and tumor progression in preclinical models (Long et al., 2019). *S. gallolyticus* has also demonstrated tumor-promoting properties in experimental CRC models, complementing its long-recognized clinical association with colorectal neoplasia (Kumar et al., 2017).

Conversely, several commensal microorganisms have been associated with epithelial-barrier maintenance and immunoregulatory activity. Species such as *Faecalibacterium prausnitzii*, *Akkermansia muciniphila*, and *Clostridium butyricum* may support mucosal homeostasis through the production of bioactive metabolites and modulation of inflammatory pathways (Chen et al., 2020; Dikeocha et al., 2022; Soheilipour et al., 2025). However, these effects are highly context dependent and should not be interpreted as uniformly protective properties of individual species. Microbial function, strain-level variation, community structure, host characteristics, and the surrounding metabolic environment may be more biologically relevant than the presence or absence of a single taxon (Zhang et al., 2019; Ternes et al., 2020).

**Figure 2:**
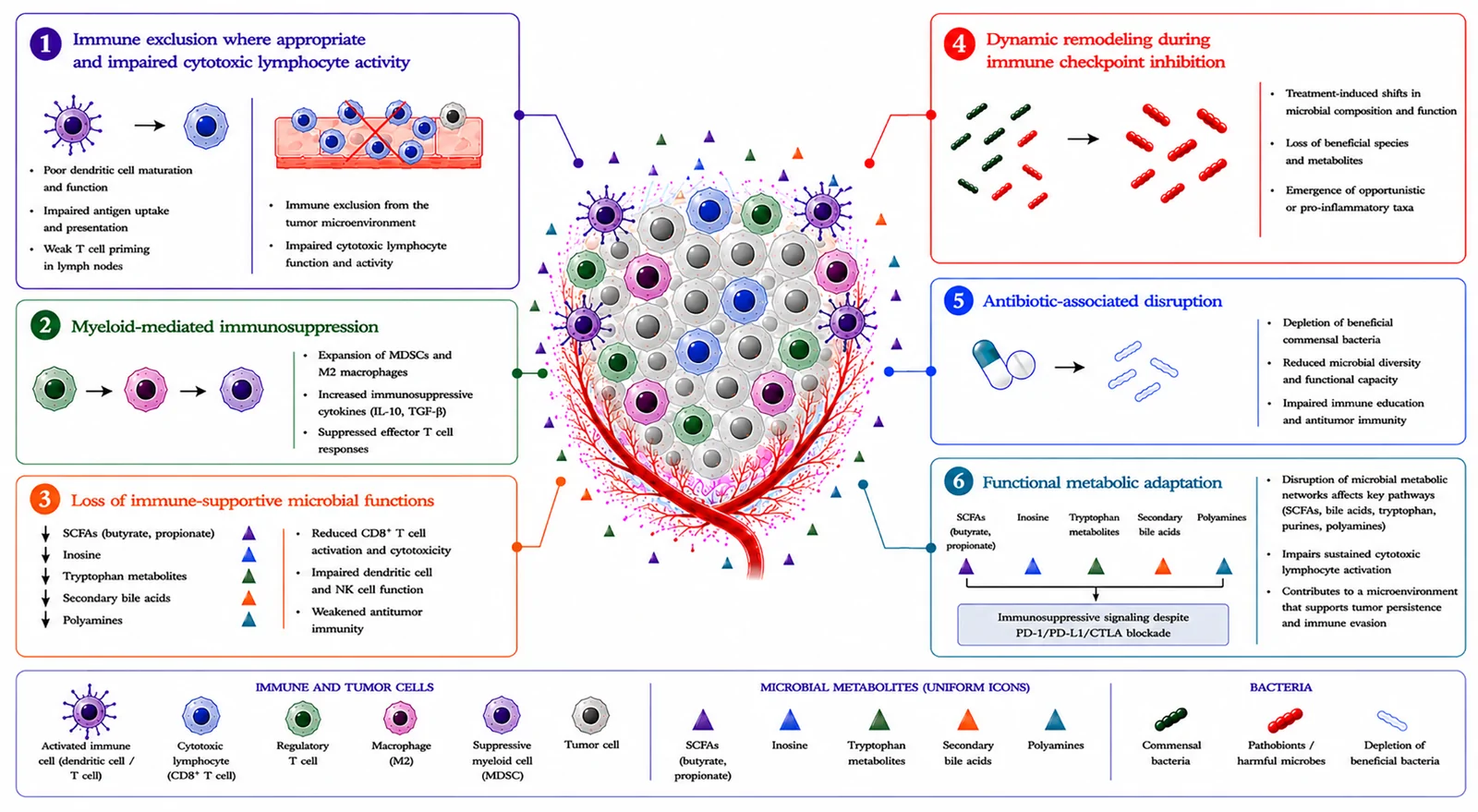
Gut microbiome-immune axis and mechanisms of primary and acquired resistance to immune checkpoint inhibitors.

Collectively, colorectal carcinogenesis is accompanied by progressive remodeling of the gut microbiome–immune axis, with alterations in microbial composition, metabolic activity, epithelial integrity, and local and systemic immunity (Wong and Yu, 2019; Ternes et al., 2020). This evolving biological environment provides a mechanistic framework through which the gut microbiome may subsequently influence sensitivity or resistance to immune checkpoint inhibition. Major microbial metabolites and their immunological effects relevant to immune checkpoint inhibition are illustrated in **Figure 2**.

### 3.3 Functional mediators of the gut microbiome–immune axis

Increasing evidence suggests that the functional characteristics of the gut microbiome may provide more biologically relevant information than taxonomic composition alone (Ternes et al., 2020). Microbial communities that differ substantially in species composition may retain overlapping metabolic capabilities, whereas closely related microbial profiles may produce different functional outputs. Accordingly, microbial genes, pathways, transcripts, proteins, and metabolites may represent more stable or mechanistically informative indicators of host–microbiome interactions than individual bacterial taxa (Zhang et al., 2019; Porcari et al., 2025).

Among the most extensively studied microbial metabolites are the short-chain fatty acids acetate, propionate, and butyrate, which are generated primarily through bacterial fermentation of dietary fiber (Tran et al., 2025). Short-chain fatty acids support epithelial-barrier integrity and influence immune-cell differentiation and function through several pathways, including activation of G protein-coupled receptors and inhibition of histone deacetylases (Luu et al., 2021). They can promote regulatory T-cell differentiation and modulate dendritic cells, macrophages, and effector T cells; however, their net effects on antitumor immunity depend on metabolite concentration, anatomical compartment, immune context, and tumor type (He et al., 2021).

Microbial metabolism of dietary tryptophan generates a diverse group of bioactive indole derivatives, including indole-3-acetic acid, indole-3-propionic acid, indole-3-aldehyde, indoleacrylic acid, tryptamine, and skatole (Roager and Licht, 2018). Several of these metabolites function as ligands of the aryl hydrocarbon receptor and may regulate epithelial-barrier function, mucosal immune homeostasis, dendritic-cell activity, and T-cell differentiation (Tran et al., 2025). These microbial pathways should be distinguished from host-mediated tryptophan catabolism through indoleamine 2,3-dioxygenase 1 (IDO1), which generates kynurenine and is commonly associated with immunosuppressive signaling within the tumor microenvironment (Pallotta et al., 2022). Alterations in the balance between microbial indole production and host kynurenine metabolism may therefore influence antitumor immunity, although direct evidence in MSI-H/dMMR CRC remains limited.

Bile acid metabolism represents another major interface between the gut microbiome and host immunity. Following hepatic synthesis, primary bile acids undergo microbial deconjugation and biotransformation into a heterogeneous group of secondary bile acids. These metabolites interact with host receptors, including the farnesoid X receptor and Takeda G protein-coupled receptor 5, and can influence epithelial integrity, macrophage polarization, inflammatory cytokine production, and adaptive immune responses (Su et al., 2023). Importantly, individual bile acid species may exert distinct and occasionally opposing immunological effects (Lin et al., 2026). Bile acids should therefore be evaluated at the level of specific metabolites, concentrations, receptors, and biological contexts rather than being interpreted as a uniform functional class.

Inosine further illustrates the context-dependent effects of microbial metabolites on antitumor immunity. Preclinical evidence indicates that microbiome-derived inosine can enhance T-cell activation and improve responses to immune checkpoint inhibition under specific conditions, particularly when appropriate co-stimulatory signals are present (Mager et al., 2020). In other settings, inosine may serve predominantly as an alternative energy substrate or exert different immunological effects (Wang et al., 2020). Its relevance as a biomarker or therapeutic mediator in colorectal cancer therefore remains investigational.

Polyamines, including putrescine, spermidine, and spermine, are derived from both host and microbial metabolism and participate in epithelial renewal, cellular proliferation, nucleic-acid stabilization, and immune regulation (Tofalo et al., 2019). Their biological effects are similarly context dependent: physiological polyamine concentrations contribute to tissue homeostasis, whereas dysregulated polyamine metabolism may support tumor growth and immunosuppressive processes (Zhu et al., 2025). The contribution of microbiome-derived polyamines to immune checkpoint inhibitor response in MSI-H/dMMR colorectal cancer remains insufficiently characterized.

Collectively, these pathways demonstrate that the gut microbiome influences antitumor immunity through coordinated metabolic and immunological signaling rather than through the activity of individual microorganisms alone. Understanding the interactions among microbial functions, host metabolism, immune-cell states, and the tumor microenvironment provides the mechanistic foundation for evaluating microbiome-mediated primary and acquired resistance to immune checkpoint inhibition.

## 4 Immune checkpoint inhibition in MSI-H/dMMR colorectal cancer: clinical success and remaining challenges

### 4.1 Biological rationale for immune checkpoint inhibition

Deficient mismatch repair (dMMR) results from the loss of function of one or more DNA mismatch repair proteins, leading to the accumulation of insertion and deletion mutations within microsatellite regions and the development of microsatellite instability-high (MSI-H) tumors (Boland and Goel, 2010). This hypermutated phenotype generates a large repertoire of neoantigens that enhances tumor immunogenicity, promotes infiltration of cytotoxic CD8^+^ T lymphocytes, and stimulates interferon-γ-driven inflammatory signaling within the tumor microenvironment (Llosa et al., 2015; Le et al., 2017). Consequently, dMMR/MSI-H colorectal cancers typically exhibit an inflamed immune phenotype characterized by abundant tumor-infiltrating lymphocytes and increased expression of multiple immune checkpoint molecules, including programmed death-ligand 1 (PD-L1), thereby providing a strong biological rationale for immune checkpoint blockade (Le et al., 2017; Overman et al., 2017; André et al., 2020).

Despite their immunogenicity, dMMR/MSI-H tumors simultaneously develop compensatory mechanisms that limit effective antitumor immunity. Persistent antigen exposure promotes progressive T-cell dysfunction and sustained expression of inhibitory receptors such as programmed cell death protein 1 (PD-1), lymphocyte activation gene-3 (LAG-3), and T-cell immunoglobulin and mucin domain-containing protein-3 (TIM-3) (van der Leun et al., 2020). In parallel, regulatory T cells (Tregs), myeloid-derived suppressor cells (MDSCs), tumor-associated macrophages (TAMs), immunosuppressive cytokines, and metabolic alterations within the tumor microenvironment further attenuate cytotoxic immune responses (Llosa et al., 2015; Binnewies et al., 2018). These adaptive immune-evasion mechanisms explain why highly immunogenic tumors may nevertheless fail to generate durable antitumor immunity and provide the biological basis for therapeutic immune checkpoint inhibition (Chen and Mellman, 2017).

### 4.2 Clinical evolution of immune checkpoint inhibitors in MSI-H/dMMR colorectal cancer

The introduction of immune checkpoint inhibitors has fundamentally transformed the management of patients with dMMR/MSI-H colorectal cancer across both metastatic and localized disease settings. Initial evidence from the phase II CheckMate-142 study demonstrated durable clinical activity of nivolumab, both as monotherapy and in combination with ipilimumab, in previously treated metastatic disease, establishing PD-1 blockade as an effective therapeutic strategy for this molecular subtype (Overman et al., 2017).

Subsequently, the phase III KEYNOTE-177 trial (André et al., 2020) established pembrolizumab as the preferred first-line treatment for metastatic dMMR/MSI-H colorectal cancer after demonstrating significantly prolonged progression-free survival, fewer treatment-related adverse events, and improved health-related quality of life compared with standard chemotherapy. More recently, the phase III CheckMate-8HW trial (André et al., 2025) showed superior progression-free survival with combined nivolumab and ipilimumab compared with nivolumab alone, further supporting dual PD-1/CTLA-4 blockade as an important therapeutic option for patients with metastatic disease.

Equally remarkable advances have been achieved in localized colorectal cancer. Neoadjuvant immunotherapy studies, including the NICHE and NICHE-2 trials (Chalabi et al., 2020; Chalabi et al., 2024) in colon cancer and the prospective study by Cercek and colleagues (Cercek et al., 2025) in locally advanced rectal cancer, have reported unprecedented pathological and clinical complete response rates in patients with dMMR/MSI-H tumors. These findings have raised the possibility of organ preservation and substantial treatment de-escalation in carefully selected patients, although longer follow-up and randomized evidence remain necessary before these strategies can be broadly implemented. Ongoing phase III trials, including ATOMIC and AZUR-2, are expected to further define the role of immune checkpoint inhibitors in perioperative treatment and earlier disease stages (Sinicrope et al., 2019; Starling et al., 2026).

Collectively, these studies have established immune checkpoint inhibition as the cornerstone of treatment for patients with dMMR/MSI-H colorectal cancer and represent one of the clearest examples of precision immuno-oncology successfully translating molecular biology into meaningful clinical benefit.

### 4.3 Beyond MSI-H: why does resistance still occur?

Importantly, despite these favorable immunological characteristics, approximately 25–35% of patients with metastatic dMMR/MSI-H colorectal cancer exhibit primary resistance to PD-1-based immunotherapy, indicating that additional tumor-intrinsic and host-related factors influence treatment response (André et al., 2020; André et al., 2025). These observations indicate that mismatch repair deficiency, although an essential predictive biomarker, is insufficient to fully explain clinical responsiveness to immune checkpoint blockade.

The mechanisms underlying resistance are complex and multifactorial, involving both tumor-intrinsic and host-related factors (Sharma et al., 2017). Tumor-intrinsic mechanisms include impaired antigen presentation through alterations in the HLA class I pathway or β2-microglobulin loss, defects in interferon-γ signaling, activation of alternative oncogenic pathways, and compensatory upregulation of additional inhibitory immune checkpoints (Blank et al., 2016; Kalbasi and Ribas, 2020). However, these mechanisms alone do not adequately explain the considerable interpatient variability observed in clinical practice, suggesting that systemic determinants of immune competence also contribute to treatment outcomes.

Among these host-related factors, the gut microbiome–immune axis has emerged as one of the most compelling modulators of immune checkpoint inhibitor efficacy. Through coordinated interactions among microbial communities, microbial metabolites, epithelial-barrier integrity, innate immunity, and adaptive immune responses, the intestinal microbiome may influence both the initiation and maintenance of effective antitumor immunity (Odenwald and Turner, 2017; Routy et al., 2018; Iliev et al., 2025; Tran et al., 2025). These interactions provide a biologically plausible explanation for why patients with apparently similar tumor genomic profiles may experience markedly different responses to immunotherapy.

Although accumulating evidence supports a role for the gut microbiome in modulating immune checkpoint inhibitor efficacy, most mechanistic data derive from preclinical models or studies in melanoma, non-small cell lung cancer, and renal cell carcinoma (Gopalakrishnan et al., 2018; Matson et al., 2018; Routy et al., 2018; Jin et al., 2022). Direct prospective evidence in patients with MSI-H/dMMR colorectal cancer remains comparatively limited, and the precise microbial, metabolic, and immunological determinants of treatment resistance have yet to be fully defined (Serpas Higbie et al., 2022; Cheng et al., 2024). The following sections therefore examine the current evidence supporting microbiome-mediated primary and acquired resistance to immune checkpoint inhibition, while emphasizing the distinction between established findings and biologically plausible, but as yet incompletely validated, mechanisms.

## 5 Gut microbiome–mediated resistance to immune checkpoint inhibition

### 5.1 Functional alterations of the gut microbiome–immune axis

As mentioned earlier numerous studies have associated specific bacterial taxa with responsiveness to immune checkpoint inhibitors, accumulating evidence suggests that alterations in microbial function may provide a more biologically meaningful explanation for treatment resistance than changes in taxonomic composition alone. Functional disturbances in microbial metabolism can influence epithelial barrier integrity, antigen presentation, immune-cell activation, and cytokine signaling through coordinated effects on microbial metabolites and host immune pathways. Consequently, alterations in short-chain fatty acid production, bile acid metabolism, tryptophan catabolism, purine metabolism, and polyamine homeostasis have emerged as plausible mechanisms contributing to primary and acquired resistance to immune checkpoint inhibition. However, the strength of evidence varies considerably among these pathways, with most mechanistic data originating from preclinical models and relatively few studies conducted specifically in patients with MSI-H/dMMR colorectal cancer (Zhuo et al., 2019; Zhang et al., 2021; Lu et al., 2023). Many of the proposed mechanisms have yet to be validated in CRC-specific systems or prospective clinical cohorts.

#### 5.1.1 Altered short-chain fatty acid signaling

In colorectal cancer, reduced abundance of butyrate-producing commensals, including *Faecalibacterium prausnitzii*, *Roseburia intestinalis* and *Eubacterium rectale*, have been associated with diminished butyrate availability and impaired antitumor immune responses in experimental models (Yachida et al., 2019). Although not a major butyrate producer itself, *Akkermansia muciniphila* may further support antitumor immunity by degrading host mucin to generate acetate and propionate, which serve as substrates for butyrate-producing commensals through metabolic cross-feeding, while simultaneously promoting epithelial barrier integrity and immune activation (Belzer and de Vos, 2012). Similarly, dietary pectin supplementation enhanced anti-PD-1 efficacy in microbiota-humanized murine CRC models, with increased butyrate production identified as an important mediator of improved CD8^+^ T-cell infiltration and activation (Zhang et al., 2021).

Despite these encouraging findings, current evidence does not support SCFAs as validated predictive biomarkers of immunotherapy response. Most available data derive from murine models, ex vivo systems, or colorectal cancer models that do not specifically focus on patients with MSI-H/dMMR disease, while prospective clinical studies specifically evaluating patients with MSI-H/dMMR disease remain lacking. Consequently, impaired SCFA signaling should currently be regarded as a biologically plausible resistance mechanism supported by substantial preclinical and translational evidence rather than an established determinant of immune checkpoint inhibitor efficacy.

#### 5.1.2 Altered bile acid signaling

Experimental evidence from colorectal cancer models suggests that individual microbiota-derived bile acids can directly modulate antitumor immune responses. Deoxycholic acid (DCA) for example, has been shown to impair cytotoxic CD8^+^ T-cell effector function by reducing the production of granzyme B, interferon-γ, and tumor necrosis factor-α, thereby promoting a more immunosuppressive tumor microenvironment (Cong et al., 2024). Although these findings provide mechanistic support for a role of bile acid metabolism in colorectal cancer immunity, they have not been demonstrated specifically in MSI-H/dMMR tumors or during immune checkpoint inhibitor therapy.

Importantly, bile acids should not be regarded as a uniformly immunosuppressive metabolite class. Ursodeoxycholic acid (UDCA) and tauroursodeoxycholic acid (TUDCA) generally exhibit epithelial-protective and anti-inflammatory properties, whereas LCA and several of its derivatives may exert divergent immunological effects depending on their concentration, receptor engagement, tissue compartment, and local immune context (Su et al., 2023). Furthermore, structurally related bile acids may activate distinct receptor networks and downstream signaling pathways, highlighting the complexity of bile acid-mediated immune regulation (Tran et al., 2025). Consequently, interpretation of bile acid signaling requires evaluation at the level of individual metabolites rather than broad classification into primary and secondary bile acids.

The frequently cited CXCL16–CXCR6 natural killer T-cell axis also warrants cautious interpretation. This mechanism was originally demonstrated in experimental liver tumor models, in which microbiome-dependent alterations in bile acid metabolism regulated hepatic NKT-cell recruitment through CXCL16 expression (Ma et al., 2018). Although biologically compelling, this pathway has not been directly validated in colorectal cancer liver metastases, MSI-H/dMMR colorectal cancer, or in the context of immune checkpoint inhibitor resistance. It should therefore be considered a hypothesis-generating mechanism rather than established evidence in colorectal cancer immunotherapy.

Overall, current evidence supports an important role for bile acid metabolism in shaping antitumor immunity, but the relationship between specific bile acid signatures and resistance to ICI remains largely inferential. Future longitudinal studies integrating targeted bile acid metabolomics with microbiome profiling and immune phenotyping in patients with MSI-H/dMMR colorectal cancer will be essential to determine whether distinct bile acid profiles can serve as predictive biomarkers or therapeutic targets for overcoming immunotherapy resistance.

#### 5.1.3 Altered tryptophan metabolism

Altered tryptophan metabolism represents another potential mechanism through which the gut microbiome may contribute to an immunological environment that favors immune evasion and reduces the efficacy of antitumor immune responses. Microbial catabolism of dietary tryptophan produces numerous indole derivatives, many of which function as ligands for the aryl hydrocarbon receptor (AhR) (Roager and Licht, 2018). Activation of AhR influences epithelial-barrier integrity, dendritic-cell maturation, innate lymphoid cell function, and the balance between regulatory and effector T-cell responses, thereby contributing to intestinal immune homeostasis. Dysbiosis-associated reductions in microbial indole production may therefore impair these immunoregulatory mechanisms and promote a less favorable environment for effective antitumor immunity.

In parallel, host-mediated tryptophan catabolism through indoleamine 2,3-dioxygenase 1 (IDO1) generates kynurenine, an immunoregulatory metabolite that suppresses cytotoxic T-cell activity while promoting regulatory T-cell differentiation and immune tolerance (Pallotta et al., 2022). Increased IDO1 expression has been associated with immune evasion in colorectal cancer (Xi et al., 2026), and experimental studies have demonstrated that enhanced IDO1 activity can reduce sensitivity to PD-1 blockade through accumulation of kynurenine and suppression of antitumor immune responses (Wu and Zhu, 2021). Although these findings implicate the IDO1–kynurenine axis as a potential contributor to immunotherapy resistance, clinical trials evaluating IDO1 inhibition in combination with immune checkpoint blockade have thus far yielded disappointing results in several malignancies, suggesting that this pathway alone is unlikely to account for resistance mechanisms (Komiya and Huang, 2018).

Overall, dysregulated tryptophan metabolism represents a biologically plausible mechanism of microbiome-mediated resistance to immune checkpoint inhibition. Future studies integrating metagenomics, metabolomics, and longitudinal immune profiling will be necessary to clarify the relative contributions of microbial indole production and host IDO1–kynurenine signaling to treatment response and resistance in MSI-H/dMMR colorectal cancer.

#### 5.1.4 Altered inosine signaling

Inosine has emerged as a promising microbiome-derived metabolite capable of modulating antitumor immunity and influencing responses to immune checkpoint inhibition. Produced through microbial purine metabolism, inosine functions not only as a metabolic intermediate but also as an immunoregulatory signaling molecule capable of enhancing T-cell activation under appropriate immunological conditions.

The pivotal study by Mager et al. (2020) demonstrated that microbiome-derived inosine enhanced the efficacy of CTLA-4 blockade in preclinical tumor models by promoting T-helper 1 (Th1) lymphocyte differentiation and augmenting cytotoxic T-cell responses through adenosine receptor-dependent signaling, provided that adequate co-stimulatory signals were present. These findings highlighted that the immunological effects of inosine are highly context dependent and cannot be interpreted independently of the surrounding cytokine milieu and antigen-presenting cell activation. Rather than acting as a universal immune stimulant, inosine appears to amplify pre-existing antitumor immune responses under favorable immunological conditions.

Although these observations have generated considerable interest, direct evidence linking altered inosine metabolism to immune checkpoint inhibitor resistance in colorectal cancer remains limited. Most available data derive from preclinical models or studies conducted in tumor types other than colorectal cancer, and prospective validation in patients with MSI-H/dMMR disease has not yet been performed (Porcari et al., 2025). Furthermore, the intestinal microbial communities responsible for inosine production vary substantially among individuals, suggesting that functional metabolic capacity may be more informative than the abundance of individual bacterial taxa.

Consequently, altered microbial inosine production should currently be regarded as a biologically plausible contributor to variability in immune checkpoint inhibitor response rather than an established mechanism of primary or acquired resistance. Future studies integrating metagenomic sequencing with targeted purine metabolomics and longitudinal immune profiling will be required to determine whether inosine metabolism can serve as a predictive biomarker or therapeutic target in patients with MSI-H/dMMR colorectal cancer.

#### 5.1.5 Dysregulated polyamine metabolism

Among the major polyamines, spermidine has attracted particular attention because of its immunomodulatory properties. Experimental studies have demonstrated that spermidine can enhance mitochondrial function, autophagy, and metabolic reprogramming of CD8^+^ T cells, thereby supporting T-cell persistence and effector function during chronic antigen exposure (Gabrilovich, 2017). These observations suggest that adequate polyamine availability may contribute to the maintenance of effective antitumor immune responses, although this relationship appears to depend on the metabolic and immunological context.

Conversely, dysregulated polyamine metabolism within the tumor microenvironment has been associated with immune suppression through multiple mechanisms, including impaired T-cell function, expansion of immunosuppressive myeloid populations, and macrophage polarization toward tumor-promoting phenotypes (Tofalo et al., 2019; Tran et al., 2025). Given that both tumor cells and infiltrating immune cells actively utilize polyamines, alterations in polyamine metabolism likely reflect complex metabolic interactions between the host, the tumor, and the gut microbiome rather than the activity of a single cellular compartment.

Despite these mechanistic insights, direct evidence linking microbiome-derived polyamines to immune checkpoint inhibitor resistance in colorectal cancer remains scarce. Most available data originate from experimental systems or broader studies of cancer immunometabolism. Altered polyamine metabolism should currently be regarded as an emerging hypothesis-generating mechanism of resistance that requires further validation before its clinical significance can be established.

### 5.2 Primary (intrinsic) resistance

Primary (intrinsic) resistance refers to the failure to achieve an initial clinical response to immune checkpoint inhibitors despite appropriate treatment, indicating that pre-existing biological factors prevent the development of effective antitumor immunity. In MSI-H/dMMR colorectal cancer, where most patients derive substantial benefit from PD-1 blockade, accumulating evidence suggests that, in addition to tumor-intrinsic mechanisms, alterations in the gut microbiome may influence the immune microenvironment and contribute to resistance in a subset of patients.

#### 5.2.1 Immune exclusion and impaired cytotoxic lymphocyte activity

The gut microbiome contributes to the establishment and maintenance of effective antitumor immunity by regulating the recruitment, activation, and persistence of cytotoxic lymphocytes within the tumor microenvironment. Through coordinated interactions involving microbial metabolites, antigen-presenting cells, and epithelial signaling, commensal microorganisms promote dendritic-cell maturation, efficient antigen presentation, and the priming of tumor-specific CD8^+^ T cells (Belkaid and Harrison, 2017; Ternes et al., 2020; Iliev et al., 2025). Dysbiosis may disrupt these processes, resulting in impaired lymphocyte recruitment, reduced T-cell infiltration, and diminished cytotoxic activity despite the high neoantigen burden characteristic of MSI-H/dMMR colorectal cancer (Llosa et al., 2015; Luu et al., 2021). Experimental studies further demonstrate that microbial metabolites, including short-chain fatty acids, inosine, and spermidine, support T-cell metabolic fitness, cytokine production, and resistance to exhaustion, whereas alterations of these pathways may compromise durable responses to immune checkpoint inhibition (Tofalo et al., 2019; Mager et al., 2020; Zhang et al., 2021). Collectively, these observations suggest that microbiome-associated immune exclusion and impaired cytotoxic lymphocyte function represent plausible contributors to primary resistance, although direct evidence in MSI-H/dMMR colorectal cancer remains limited.

#### 5.2.2 Myeloid-mediated immunosuppression

The gut microbiome also influences antitumor immunity through its effects on myeloid-cell populations within the tumor microenvironment. Microbial-derived metabolites and inflammatory mediators regulate macrophage polarization, dendritic-cell function, and the accumulation of myeloid-derived suppressor cells (MDSCs), thereby shaping the balance between immune activation and immune suppression (Gabrilovich, 2017). Dysbiosis may promote expansion of immunosuppressive myeloid populations that inhibit T-cell activation through cytokine secretion, nutrient depletion, production of reactive oxygen and nitrogen species, and expression of inhibitory ligands (Cong et al., 2024). In parallel, altered microbial signaling may impair dendritic-cell maturation and antigen presentation, further limiting effective adaptive immune responses. Although these mechanisms have been extensively characterized in experimental cancer models, their precise contribution to immune checkpoint inhibitor resistance in MSI-H/dMMR colorectal cancer remains incompletely defined. Nevertheless, the cumulative evidence supports myeloid-cell reprogramming as an important component of microbiome-mediated intrisic resistance to ICI.

#### 5.2.3 Loss of immune-supportive microbial functions

Emerging evidence suggests that the loss of beneficial microbial functions may be more relevant to immunotherapy resistance than the expansion of individual pathogenic microorganisms (Odenwald and Turner, 2017; Tran et al., 2025). Rather than acting through specific bacterial species, the gut microbiome maintains immune competence through coordinated metabolic activities, including short-chain fatty acid production, bile acid biotransformation, microbial tryptophan metabolism, purine metabolism, and polyamine biosynthesis. Disruption of these functional networks may impair epithelial-barrier integrity, alter cytokine signaling, reduce antigen presentation, and compromise T-cell activation, collectively creating an immune milieu that is less responsive to immune checkpoint blockade (Tofalo et al., 2019; Mager et al., 2020; Zhang et al., 2021). This functional perspective may also explain the limited reproducibility of taxonomic biomarkers across independent studies, as distinct microbial communities can exhibit overlapping metabolic capabilities while similar taxonomic profiles may differ substantially in functional output. Consequently, functional metagenomics, transcriptomics, and metabolomics are increasingly regarded as more informative approaches for characterizing microbiome-associated resistance than taxonomic profiling alone (Porcari et al., 2025). Although these concepts remain under active investigation, they provide a biologically coherent framework linking microbial dysbiosis with impaired antitumor immunity and subsequent resistance to immune checkpoint inhibition.

### 5.3 Acquired resistance

Unlike primary resistance, which is present before treatment initiation, acquired resistance develops after an initial clinical response to immune checkpoint inhibitors and remains a major obstacle to durable disease control in patients with MSI-H/dMMR colorectal cancer (Sharma et al., 2017; Stemmer et al., 2024). Although tumor-intrinsic mechanisms, including immune editing, defects in antigen presentation, and alterations in interferon signaling, are well-established contributors to acquired resistance, the potential role of the gut microbiome is considerably less well understood. Current evidence derives predominantly from preclinical models and observational studies, while longitudinal data in colorectal cancer remain limited. Consequently, microbiome-mediated acquired resistance should currently be regarded as a biologically plausible but incompletely validated mechanism that warrants further investigation.

#### 5.3.1 Dynamic remodeling during immune checkpoint inhibition

The gut microbiome is a dynamic ecosystem that continuously adapts to changes in the host environment throughout cancer therapy. Longitudinal studies suggest that immune checkpoint inhibition itself may influence microbial composition and function through reciprocal interactions between systemic immunity, mucosal immune responses, epithelial barrier integrity, and microbial ecology (Fidelle et al., 2023; Zeng et al., 2023). As treatment progresses, alterations in microbial metabolic activity may gradually reshape the tumor immune microenvironment, potentially contributing to diminished therapeutic efficacy despite continued checkpoint blockade (Porcari et al., 2025). Rather than reflecting the emergence or disappearance of individual bacterial species, these changes are increasingly thought to involve shifts in microbial functional capacity, although direct evidence in MSI-H/dMMR colorectal cancer remains scarce. Prospective longitudinal studies integrating serial microbiome sampling with immune profiling will be necessary to determine whether these microbial alterations precede clinical progression or simply accompany tumor evolution.

#### 5.3.2 Antibiotic-associated disruption

Exposure to antibiotics represents one of the most extensively studied external modifiers of the gut microbiome during immunotherapy. Broad-spectrum antimicrobial therapy may reduce microbial diversity, disrupt immune-supportive microbial functions, and alter the production of metabolites involved in antitumor immunity (Crespin et al., 2023). Several retrospective studies across multiple tumor types have reported associations between recent antibiotic exposure and inferior outcomes following immune checkpoint inhibition, although the magnitude and consistency of these effects vary considerably between studies (Jin et al., 2019; Derosa et al., 2020; Crespin et al., 2023). Interpretation remains challenging because antibiotic administration is frequently linked to infection, advanced disease, hospitalization, or declining clinical status, all of which may independently influence survival. Comparable data in MSI-H/dMMR colorectal cancer are limited and current evidence does not establish a direct causal relationship between antibiotic-induced dysbiosis and acquired resistance (Serpas Higbie et al., 2022). Nevertheless, these observations highlight the importance of antimicrobial stewardship and careful interpretation of microbiome-related clinical studies.

#### 5.3.3 Functional metabolic adaptation

An emerging hypothesis proposes that acquired resistance may result from progressive disruption of microbial metabolic networks rather than irreversible changes in microbial taxonomy. Functional alterations affecting short-chain fatty acid production, bile acid biotransformation, tryptophan metabolism, purine metabolism, and polyamine homeostasis may also impair sustained activation of cytotoxic lymphocytes, promote immune exhaustion, and reinforce immunosuppressive signaling despite continued PD-1 or PD-L1 blockade (Tofalo et al., 2019; Mager et al., 2020; Zhang et al., 2021; Su et al., 2023; Tran et al., 2025). Chronic immune activation during treatment may further modify cytokine networks, epithelial barrier function, and mucosal immunity, creating bidirectional interactions between the evolving tumor, host immune system, and gut microbiome (Zhang et al., 2019; Porcari et al., 2025). Although these concepts are supported by experimental evidence and remain biologically plausible, direct clinical validation in MSI-H/dMMR colorectal cancer is currently lacking. Future studies combining longitudinal metagenomic, metabolomic, and immune profiling will be essential to determine whether dynamic alterations in microbial function contribute to acquired resistance and whether these pathways can be therapeutically targeted to prolong the durability of immune checkpoint inhibition.

## 6 Biomarkers of the gut microbiome–immune axis

Although numerous studies have identified associations between the gut microbiome and clinical outcomes following immune checkpoint inhibition, translation into clinically applicable biomarkers has proven challenging. Most candidate biomarkers remain investigational owing to substantial interindividual variability, differences in sequencing methodologies, geographic and dietary influences, and the dynamic nature of the gut microbiome. Contemporary biomarker development is shifting toward integrated approaches that combine microbial, host, immunological, and metabolic information.

### 6.1 Functional biomarkers

Early biomarker studies primarily focused on identifying individual bacterial taxa associated with response to immune checkpoint inhibitors (Gopalakrishnan et al., 2018; Derosa et al., 2020; Jin et al., 2022). However, considerable variability between cohorts has limited the reproducibility and clinical applicability of taxonomic biomarkers (Knight et al., 2018; Limeta et al., 2020). Distinct microbial communities may perform similar metabolic functions, whereas apparently comparable microbial compositions can differ substantially in functional activity. Consequently, functional biomarkers are increasingly regarded as more informative indicators of microbiome-mediated immune regulation.

Among the most promising candidates are microbial metabolic signatures that directly influence epithelial barrier integrity, antigen presentation, T-cell activation, cytokine production, and immune-cell metabolism, thereby providing mechanistic links between the gut microbiome and antitumor immunity. Advances in metagenomic, metatranscriptomic, metaproteomic, and metabolomic technologies now enable characterization of these functional pathways with substantially greater biological resolution than conventional 16S rRNA sequencing (Knight et al., 2018; Tremblay et al., 2022).

Nevertheless, functional biomarkers also face important limitations. Microbial metabolic activity is highly dynamic and may be influenced by diet, medications, antibiotic exposure, host metabolism, and disease progression. Standardization of sample collection, analytical pipelines, metabolite quantification, and bioinformatic interpretation therefore remains essential before functional microbiome biomarkers can be incorporated into routine clinical practice.

### 6.2 Integrated host–microbiome biomarkers

The complexity of antitumor immunity suggests that no single biomarker is likely to accurately predict response to immune checkpoint inhibition. Instead, integrated biomarker models combining microbial and host-derived variables may provide superior predictive performance. Such approaches incorporate microbial functional profiles alongside established tumor biomarkers, including mismatch repair status, tumor mutational burden, PD-L1 expression, immune-cell infiltration, circulating inflammatory markers, cytokine profiles, and genomic or transcriptomic signatures (Blank et al., 2016; Sharma et al., 2017).

Recent studies increasingly employ multi-omic approaches that integrate metagenomics, metabolomics, transcriptomics, proteomics, and immunophenotyping to characterize the bidirectional interactions between the gut microbiome and host immunity (Zhu et al., 2025). Machine learning algorithms are further facilitating identification of complex biomarker signatures that outperform individual microbial taxa or isolated clinical variables (Novielli et al., 2025). Although these approaches remain largely investigational, they may better capture the multifactorial biological processes governing immunotherapy responsiveness.

For patients with MSI-H/dMMR colorectal cancer, integrated biomarker models may ultimately improve patient stratification by identifying individuals at increased risk of primary or acquired resistance despite otherwise favorable molecular characteristics. However, prospective validation in large, well-annotated clinical cohorts remains necessary before these models can inform therapeutic decision-making.

### 6.3 Emerging technologies and future directions in biomarker development

Rapid advances in sequencing technologies and systems biology are transforming the field of microbiome biomarker discovery. High-resolution shotgun metagenomic sequencing, single-cell transcriptomics, spatial transcriptomics, metabolomics, and computational multi-omic integration now permit comprehensive characterization of host–microbiome interactions at unprecedented depth (Papalexi and Satija, 2018; Zhang et al., 2019; Marx, 2021; Tremblay et al., 2022). These technologies are shifting research beyond descriptive taxonomic profiling toward functional characterization of microbial ecosystems and their influence on antitumor immunity.

Longitudinal sampling represents another important direction for biomarker development. Because the gut microbiome is dynamic, serial assessment before and during immune checkpoint inhibition may provide greater predictive value than single baseline measurements by capturing temporal changes associated with treatment response or emerging resistance (Zeng et al., 2023). Integration of longitudinal microbiome analyses with circulating biomarkers, including circulating tumor DNA, immune-cell phenotyping, cytokine profiling, and metabolic signatures, may further improve the identification of patients likely to benefit from immunotherapy (Finotello and Trajanoski, 2018).

Despite these advances, several challenges remain. Variability in sample acquisition, sequencing platforms, bioinformatic pipelines, dietary habits, medication exposure, and geographic factors continues to limit reproducibility across studies. Moreover, most available evidence is derived from melanoma and other solid tumors, whereas prospective validation in MSI-H/dMMR colorectal cancer remains limited. Future biomarker development will therefore require standardized methodologies, international collaborative cohorts, and harmonized analytical frameworks. Ultimately, clinically useful biomarkers are likely to integrate microbial function, host immunity, tumor biology, and longitudinal disease dynamics rather than relying on individual microbial taxa alone.

Representative taxonomic, functional, and integrated microbiome biomarkers currently under investigation for predicting immune checkpoint inhibitor response are summarized in **Table 1**.

**Table 1:** Microbiome-derived biomarkers of response to immune checkpoint inhibitors in MSI-H/dMMR colorectal cancer.

Biomarker	Biological rationale	Representative evidence	Advantages	Limitations	Clinical readiness
Akkermansia muciniphila	Enhanced antigen presentation, epithelial integrity, and T-cell activation	Preclinical + early clinical	Strong biological rationale	Variable abundance across cohorts	Experimental
Faecalibacterium prausnitzii	Butyrate production and epithelial-barrier maintenance	Early clinical	Mechanistic support	Influenced by diet and geography	Experimental
Bifidobacterium spp.	Dendritic-cell maturation and CD8^+^ T-cell activation	Mainly preclinical	Reproducible immune effects	Strain-specific variability	Experimental
Fusobacterium nucleatum	Immune exclusion and immunosuppressive microenvironment	Clinical observational	Strong biological rationale	Not specific for ICI resistance	Early clinical
SCFAs	Functional regulation of epithelial integrity and antitumor immunity	Preclinical + translational	Functional biomarker	Concentration- and context-dependent	Experimental
Bile acid signatures	Reflect microbiome-mediated immune and metabolic regulation	Preclinical + translational	Mechanistically informative	High metabolic complexity	Experimental
Tryptophan metabolites	AhR-mediated immune regulation	Emerging	Functional readout	Limited CRC-specific data	Experimental
Inosine	Purine metabolite promoting Th1/CD8 responses under appropriate immune conditions	Preclinical	Mechanistic biomarker	Limited human validation	Experimental
Polyamine signatures	Reflect microbial and host immunometabolic interactions	Preclinical	Functional metabolic biomarker	Limited clinical validation	Experimental
Microbial metabolomic signatures	Capture microbial functional activity and metabolite production	Emerging multi-omic studies	May outperform taxonomy	Lack of methodological standardization	Exploratory

The information summarized in this table is derived from the studies discussed throughout the main text and is intended to provide an overview of the current evidence.

## 7 Therapeutic modulation of the gut microbiome–immune axis to overcome resistance to immune checkpoint inhibition

The growing recognition of the gut microbiome as a determinant of immunotherapy responsiveness has stimulated considerable interest in microbiome-directed therapeutic interventions. Rather than targeting individual bacterial species, most current strategies aim to restore immune-supportive microbial functions, improve microbial ecosystem resilience, or selectively manipulate host–microbiome interactions. However, despite encouraging preclinical findings and early clinical studies, no microbiome-directed intervention has yet been incorporated into routine clinical practice for patients with MSI-H/dMMR colorectal cancer. Most available evidence originates from melanoma and other solid tumors (Sivan et al., 2015; Vétizou et al., 2015; Baruch et al., 2021; Davar et al., 2021), while prospective colorectal cancer-specific data remain limited (Almonte et al., 2026). Consequently, current therapeutic approaches should be regarded as investigational, with future success likely depending on individualized and functionally guided microbiome modulation.

### 7.1 Broad ecosystem modulation

Broad microbiome-modulating strategies aim to improve the overall functional capacity of the intestinal ecosystem rather than introducing or eliminating specific microorganisms. These interventions seek to enhance microbial diversity, restore immune-supportive metabolic pathways, and improve epithelial and immune homeostasis. Although generally safe and biologically plausible, clinical evidence supporting their ability to enhance immune checkpoint inhibitor efficacy remains limited.

#### 7.1.1 Dietary interventions

Diet represents one of the strongest environmental determinants of gut microbial composition and function. Diets rich in dietary fiber, whole grains, legumes, fruits, and vegetables promote microbial diversity and increase production of short-chain fatty acids that support epithelial integrity and antitumor immunity (Spencer et al., 2021). In contrast, Western dietary patterns characterized by high intake of saturated fats, refined carbohydrates, and ultra-processed foods have been associated with reduced microbial diversity and alterations in microbial metabolism (Nicholson et al., 2012). Observational studies suggest that higher dietary fiber intake may correlate with improved outcomes following immune checkpoint inhibition, although causality has not been established and randomized interventional trials remain limited (Spencer et al., 2021). Dietary modification therefore represents an attractive, low-risk strategy for supporting immune-compatible microbial ecosystems, but its role as an adjunct to immunotherapy requires further prospective evaluation.

#### 7.1.2 Prebiotics

Prebiotics are non-digestible dietary substrates that selectively stimulate the growth and metabolic activity of beneficial microorganisms. Compounds such as inulin, resistant starch, fructooligosaccharides, and galactooligosaccharides promote production of short-chain fatty acids and other metabolites involved in immune regulation (Ghali et al., 2024; Somodi et al., 2025). Rather than directly altering bacterial composition, prebiotics may preferentially enhance immune-supportive microbial functions. Nevertheless, interindividual variability in baseline microbiome composition may substantially influence therapeutic responses, and robust clinical evidence demonstrating improved immunotherapy outcomes remains lacking.

#### 7.1.3 Probiotics

Probiotics have attracted considerable interest as potential modulators of antitumor immunity. However, commercially available probiotic formulations typically contain a limited number of bacterial strains and may not adequately restore the complexity of disrupted microbial ecosystems. Furthermore, a study in melanoma patients receiving ICI suggested that indiscriminate probiotic administration following antibiotic exposure may delay recovery of the native microbiome (Spencer et al., 2021). Although recent preclinical studies have identified specific probiotic candidates, most notably *Clostridium butyricum*, that enhance anti-PD-1 activity in colorectal cancer models (Xie et al., 2025), these findings have not yet been validated in prospective clinical trials. Current evidence supporting probiotic use during immune checkpoint inhibition therefore remains insufficient, and routine probiotic supplementation cannot currently be recommended solely to enhance immunotherapy efficacy in patients with MSI-H/dMMR colorectal cancer.

#### 7.1.4 Synbiotics and postbiotics

Synbiotics are described as combination of probiotic bacteria with complementary prebiotic substrates intended to enhance survival, colonization, and metabolic activity of administered microorganisms. Synbiotics have been investigated primarily in the perioperative setting regarding colorectal cancer, where studies didn’t demonstrate significant difference in effect regarding postoperative infections between probiotic and synbiotic (Zeng et al., 2021; Veziant et al., 2022). However, these findings have been inconsistent across randomized trials and are complicated by substantial heterogeneity in probiotic strains, prebiotic substrates, treatment duration, and patient populations. Importantly, there is currently no clinical evidence demonstrating that synbiotics improve immune checkpoint inhibitor response or overcome resistance in MSI-H/dMMR colorectal cancer. Therefore, their potential role remains hypothetical and warrants prospective investigation.

Postbiotics have recently emerged as a novel class of microbiome-based therapeutics that may address several limitations associated with the administration of live microorganisms. According to the *International Scientific Association of Probiotics and Prebiotics* (ISAPP), postbiotics are defined as preparations of inanimate microorganisms and/or their components that confer a health benefit on the host (Salminen et al., 2021). These preparations may contain inactivated microbial cells together with their structural components, cell wall constituents, extracellular vesicles, proteins, peptides, enzymes, and other associated bioactive molecules, but they do not rely on the presence of viable microorganisms. Compared with probiotics, postbiotics offer several theoretical advantages, including improved manufacturing consistency, enhanced storage stability, standardized composition, and a lower theoretical risk of microbial translocation or infection (Kudra et al., 2023). Experimental studies suggest that postbiotic preparations can modulate epithelial barrier integrity and both innate and adaptive immune responses, supporting their potential as function-oriented microbiome therapeutics (Xie et al., 2024). However, evidence in colorectal cancer remains limited, and most available data originate from experimental models (Chuah et al., 2019; Tukenmez et al., 2019; Zhong et al., 2024). To date, no preclinical or clinical studies have demonstrated that postbiotics improve immune checkpoint inhibitor efficacy or overcome primary or acquired resistance in patients with MSI-H/dMMR colorectal cancer. Consequently, although postbiotics represent a promising and highly standardized microbiome-based therapeutic platform, their role in cancer immunotherapy remains hypothetical and requires validation through dedicated translational and prospective clinical studies.

#### 7.1.5 Selective antimicrobial targeting

There has been an increasing interest in selectively eliminating microorganisms or microbial functions that promote immune suppression while preserving beneficial commensals. Potential approaches include bacteriophage therapy, narrow-spectrum antibiotics, antimicrobial peptides, CRISPR-based microbial editing, and inhibitors targeting specific microbial metabolic pathways (Elkrief et al., 2025). Compared with conventional broad-spectrum antibiotics, these strategies may allow more precise modulation of the intestinal ecosystem while minimizing collateral disruption of microbial diversity. However, most remain at the preclinical stage, and their safety, specificity, and long-term ecological consequences require further investigation before clinical implementation.

### 7.2 Fecal microbiota transplantation

Fecal microbiota transplantation (FMT) represents the most extensively investigated microbiome-directed therapeutic strategy for overcoming resistance to immune checkpoint inhibition. Early clinical studies in melanoma demonstrated that transplantation of fecal microbiota from immunotherapy responders into patients with refractory disease induced microbial engraftment, enhanced intratumoral immune activation, and restored clinical responses in a subset of patients (Baruch et al., 2021; Davar et al., 2021). These small sample observational studies provided the first proof-of-concept that modulation of the gut microbiome could influence sensitivity to immune checkpoint blockade.

Translation of this approach to colorectal cancer remains comparatively limited, but is actively expanding. The phase II RENMIN-215 trial (Zhao et al., 2023) included 20 patients with heavily pretreated, refractory microsatellite stable (MSS) metastatic CRC and evaluated FMT in combination with the PD-1 inhibitor tislelizumab and peroral anti-angiogenic agent fruquintinib. Zhao W. et al. demonstrated improved survival outcomes with manageable safety as third or later line therapy, representing a notable proof-of-concept for FMT as a chemosensitizing or immunosensitizing adjunct even in the traditionally “cold” MSS subtype. Of particular relevance to MSI-H/dMMR mCRC, a dedicated phase II trial (ClinicalTrials.gov, 2021) is investigating fecal microbiota transplantation as a resensitization strategy in this specific population. The trial design involves FMT from dMMR CRC patients who previously achieved a response to PD-1 blockade into dMMR CRC patients who failed to respond, followed by re-introduction of anti-PD-1 therapy (pembrolizumab or nivolumab). To date, this represents the only identified trial directly targeting the MSI-H/dMMR mCRC population with an FMT-based resensitization approach.

Despite these promising findings, important challenges remain before FMT can be broadly implemented. Clinical responses have been variable, donor-dependent effects appear substantial, and the mechanisms underlying successful engraftment remain incompletely understood. Furthermore, concerns regarding pathogen transmission, multidrug-resistant organisms, standardized donor screening, manufacturing consistency, regulatory oversight, and long-term safety continue to limit widespread clinical application.

### 7.3 Translational challenges

Translation of microbiome research into clinical practice is complicated by substantial biological and methodological heterogeneity. Interindividual differences in diet, geography, medications, antibiotic exposure, host genetics, and baseline microbial composition contribute to marked variability across studies. In addition, differences in stool collection, sequencing technologies, bioinformatic pipelines, metabolomic analyses, and statistical methodologies frequently limit reproducibility and cross-study comparisons (Porcari et al., 2025).

Another major challenge is distinguishing causal microbial mechanisms from associative findings. Because the gut microbiome is highly dynamic, observed microbial alterations may reflect disease progression, systemic inflammation, treatment effects, or lifestyle factors rather than directly influencing immunotherapy response. Consequently, future studies should prioritize standardized protocols, longitudinal sampling, functional multi-omic analyses, and prospective validation in well-characterized clinical cohorts. Regulatory frameworks, manufacturing standards, and quality-control procedures for microbiome-based therapeutics will also require further refinement before widespread clinical adoption.

### 7.4 Next-generation precision approaches

The future of microbiome-directed immunotherapy is likely to move beyond broad ecological manipulation toward precision modulation of microbial function. Rather than administering undefined microbial communities, emerging strategies aim to selectively engineer immune-supportive metabolic pathways through defined bacterial consortia, live biotherapeutic products, engineered microorganisms, targeted bacteriophages, synthetic microbial communities, and microbiome-derived metabolites (Elkrief et al., 2025).

Integration of metagenomics, metabolomics, transcriptomics, artificial intelligence, and systems biology may enable personalized microbiome interventions tailored to an individual’s microbial functional profile, immune phenotype, and tumor characteristics. Such approaches may identify patients most likely to benefit from microbiome modulation while allowing dynamic monitoring of treatment-induced changes during immune checkpoint inhibition. Although these technologies remain largely experimental, they represent an important conceptual shift from taxonomy-based interventions toward precision manipulation of host–microbiome interactions.

Ultimately, successful microbiome-directed therapies will likely depend on individualized strategies that integrate microbial function, host immunity, tumor biology, and longitudinal biomarker assessment rather than relying on universal interventions applicable to all patients. As mechanistic understanding continues to improve, precision microbiome engineering may become an important adjunct to immune checkpoint inhibition in MSI-H/dMMR colorectal cancer, although substantial translational and regulatory challenges remain before this vision can be realized.

Current and emerging microbiome-directed therapeutic strategies, together with their mechanisms, evidence, advantages, limitations, and stage of development, are summarized in **Table 2**.

**Table 2:** Current and emerging microbiome-directed therapeutic strategies for enhancing immune checkpoint inhibitor response in MSI-H/dMMR colorectal cancer.

Strategy	Mechanism	Current evidence	Advantages	Limitations	Development stage
Dietary intervention	Modulates microbial composition and metabolism; increases SCFA production	Observational studies and small interventional trials	Safe, inexpensive, broadly applicable	No standardized dietary protocol; limited prospective evidence	Supportive care
Prebiotics	Selectively stimulate growth and metabolic activity of beneficial microorganisms	Early clinical and preclinical studies	Low toxicity; enhances production of beneficial microbial metabolites	Variable efficacy due to baseline microbiome differences	Early clinical investigation
Probiotics	Administration of live microorganisms to modulate the gut microbiome	Mixed preclinical and early clinical evidence	Widely available; generally well tolerated	Limited evidence for improving ICI efficacy; strain-specific effects	Early clinical investigation
Synbiotics	Combination of probiotics and prebiotics to enhance microbial function	Limited clinical evidence, mainly perioperative supportive care	Potential synergistic modulation of the gut microbiome	No evidence for improving ICI efficacy; heterogeneous formulations	Early clinical investigation
Postbiotics	Preparations of inanimate microorganisms and/or their bioactive components	Predominantly preclinical evidence	Improved standardization, manufacturing consistency, and theoretical safety	Limited human data; uncertain optimal formulations	Preclinical/Early translational
Fecal microbiota transplantation (FMT)	Replacement of the intestinal microbial ecosystem using donor microbiota	Early-phase clinical trials	Strongest clinical rationale; proof-of-concept for restoring ICI sensitivity	Donor selection, engraftment variability, pathogen screening, regulatory challenges	Early clinical
Live biotherapeutic products (LBPs)	Administration of defined live bacterial consortia with therapeutic intent	Phase I/II clinical studies	Standardized composition; improved reproducibility over FMT	Limited efficacy data; manufacturing and regulatory complexity	Early clinical
Engineered microbiota	Genetically modified microorganisms designed to deliver targeted therapeutic functions	Preclinical studies	Precision modulation of microbial function and host immunity	Safety, regulatory, and manufacturing challenges	Preclinical
Bacteriophage therapy	Selective elimination or modulation of specific bacterial taxa	Preclinical studies	High specificity with minimal disruption of commensal microbiota	Very limited oncology data; resistance and delivery challenges	Preclinical

The information summarized in this table is derived from the studies discussed throughout the main text and is intended to provide an overview of the current evidence.

## 8 Future perspectives and research priorities

Despite substantial advances in understanding the relationship between the gut microbiome and immune checkpoint inhibitor efficacy, many mechanistic and translational questions remain unresolved. Current evidence supports the concept that the gut microbiome influences antitumor immunity; however, the precise microbial functions responsible for treatment response and resistance remain incompletely understood. Future research should therefore move beyond descriptive taxonomic profiling toward functional characterization of host–microbiome interactions through integrated metagenomics, metatranscriptomics, metaproteomics, and metabolomics. Compared with bacterial taxonomy alone, microbial metabolic pathways—including short-chain fatty acid production, bile acid biotransformation, tryptophan metabolism, purine metabolism, and polyamine homeostasis—are more directly linked to immune regulation and may provide more robust biomarkers of treatment responsiveness. Standardized functional profiling combined with prospective clinical validation will be essential for translating microbiome research into clinically applicable strategies for patients with MSI-H/dMMR colorectal cancer.

The complexity of host–microbiome interactions also necessitates integration of microbial functional data with tumor genomics, immune-cell phenotyping, circulating biomarkers, metabolomics, and clinical characteristics. Longitudinal sampling before, during, and after immune checkpoint inhibition will be particularly important for distinguishing baseline predictive biomarkers from dynamic microbial changes associated with treatment response, immune-related adverse events, and acquired resistance. Advances in artificial intelligence and systems biology are expected to facilitate interpretation of these highly complex multi-omic datasets, enabling identification of nonlinear relationships between microbial function, host immunity, tumor biology, and clinical outcomes that are unlikely to be captured using conventional analytical approaches. However, successful implementation of these technologies will require standardized methodologies, harmonized bioinformatic pipelines, externally validated predictive models, and large multicenter datasets to ensure reproducibility and clinical generalizability.

Future microbiome-directed therapies are likely to shift from broad ecological manipulation toward precision modulation of microbial function through defined bacterial consortia, live biotherapeutic products, engineered microorganisms, targeted bacteriophages, microbiome-derived metabolites, and other rationally designed interventions. Rather than relying on universal therapeutic strategies, successful clinical implementation will likely require individualized approaches based on microbial functional profiles, host immune characteristics, tumor biology, and longitudinal biomarker assessment. Achieving this goal will depend on prospective randomized clinical trials, standardized regulatory frameworks, improved manufacturing and quality-control processes, and a deeper mechanistic understanding of host–microbiome interactions. Ultimately, the successful translation of microbiome science into clinical oncology will require a transition from descriptive association studies toward mechanistically driven, function-oriented, and patient-specific interventions capable of improving the efficacy and durability of immune checkpoint inhibition in MSI-H/dMMR colorectal cancer.

## 9 Conclusions

The gut microbiome has emerged as an important regulator of antitumor immunity and a potential determinant of immune checkpoint inhibitor response in MSI-H/dMMR colorectal cancer. Current evidence indicates that microbial function, rather than taxonomic composition alone, is likely to underlie many of the interactions between the intestinal microbiome, the host immune system, and the tumor microenvironment. Although considerable progress has been made in elucidating these mechanisms, most microbiome-based biomarkers and therapeutic strategies remain investigational, and their clinical implementation will require rigorous prospective validation. Future advances will depend on integrating functional microbiome profiling with host and tumor biology to develop reproducible biomarkers and precision microbiome-directed interventions. A deeper mechanistic understanding of host–microbiome interactions has the potential to improve patient selection, overcome resistance to immunotherapy, and ultimately contribute to more personalized treatment strategies for colorectal cancer.

## References

[B1] Abushukair H. Ababneh O. Zaitoun S. Saeed A. (2022). Primary and secondary immune checkpoint inhibitors resistance in colorectal cancer: Key mechanisms and ways to overcome resistance. Cancer Treat. Res. Commun. 33, 100643. doi: 10.1016/j.ctarc.2022.100643 36175334

[B2] Allam-Ndoul B. Castonguay-Paradis S. Veilleux A. (2020). Gut microbiota and intestinal trans-epithelial permeability. Int. J. Mol. Sci. 21, 6402. doi: 10.3390/ijms21176402 32899147 PMC7503654

[B3] Almonte A. A. Thomas S. Iebba V. Kroemer G. Derosa L. Zitvogel L. (2026). Gut dysbiosis in oncology: a risk factor for immunoresistance. Cell Res. 36, 103–120. doi: 10.1038/s41422-025-01212-6 41535719 PMC12847903

[B4] Ambrosini M. Rousseau B. Manca P. Artz O. Marabelle A. André T. . (2024). Immune checkpoint inhibitors for POLE or POLD1 proofreading-deficient metastatic colorectal cancer. Ann. Oncol. 35, 643–655. doi: 10.1016/j.annonc.2024.03.009 38777726

[B5] André T. Elez E. Lenz H. J. Jensen L. H. Touchefeu Y. Van Cutsem E. . (2025). Nivolumab plus ipilimumab versus nivolumab in microsatellite instability-high metastatic colorectal cancer (CheckMate 8HW): a randomised, open-label, phase 3 trial. Lancet 405, 383–395. doi: 10.1016/s0140-6736(24)02848-4 39874977

[B6] André T. Shiu K. K. Kim T. W. Jensen B. V. Jensen L. H. Punt C. J. A. . (2020). Pembrolizumab in microsatellite-instability-high advanced colorectal cancer. N. Engl. J. Med. 383, 2207–2218. doi: 10.1056/NEJMoa2017699 33264544

[B7] Arthur J. C. Perez-Chanona E. Mühlbauer M. Tomkovich S. Uronis J. M. Fan T. J. . (2012). Intestinal inflammation targets cancer-inducing activity of the microbiota. Science 338, 120–123. doi: 10.1126/science.1224820 22903521 PMC3645302

[B8] Baruch E. N. Youngster I. Ben-Betzalel G. Ortenberg R. Lahat A. Katz L. . (2021). Fecal microbiota transplant promotes response in immunotherapy-refractory melanoma patients. Science 371, 602–609. doi: 10.1126/science.abb5920 33303685

[B9] Belkaid Y. Harrison O. J. (2017). Homeostatic immunity and the microbiota. Immunity 46, 562–576. doi: 10.1016/j.immuni.2017.04.008 28423337 PMC5604871

[B10] Belzer C. de Vos W. M. (2012). Microbes inside-from diversity to function: the case of Akkermansia. ISME J. 6, 1449–1458. doi: 10.1038/ismej.2012.6 22437156 PMC3401025

[B11] Binnewies M. Roberts E. W. Kersten K. Chan V. Fearon D. F. Merad M. . (2018). Understanding the tumor immune microenvironment (TIME) for effective therapy. Nat. Med. 24, 541–550. doi: 10.1038/s41591-018-0014-x 29686425 PMC5998822

[B12] Blank C. U. Haanen J. B. Ribas A. Schumacher T. N. (2016). Cancer immunology. The 'cancer immunogram'. Science 352, 658–660. doi: 10.1126/science.aaf2834 27151852

[B13] Boland C. R. Goel A. (2010). Microsatellite instability in colorectal cancer. Gastroenterology 138, 2073–2087.e3. doi: 10.1053/j.gastro.2009.12.064 20420947 PMC3037515

[B14] Cercek A. Bachet J. B. Capdevila J. Starling N. Chen E. Salvatore L. . (2025). A phase two, single-arm, open-label study with dostarlimab monotherapy in participants with untreated stage II/III dMMR/MSI-H locally advanced rectal cancer (AZUR-1). Clin. Colorectal Cancer 24, 325–330. doi: 10.1016/j.clcc.2025.02.003 40107952

[B15] Chalabi M. Fanchi L. F. Dijkstra K. K. Van den Berg J. G. Aalbers A. G. Sikorska K. . (2020). Neoadjuvant immunotherapy leads to pathological responses in MMR-proficient and MMR-deficient early-stage colon cancers. Nat. Med. 26, 566–576. doi: 10.1038/s41591-020-0805-8 32251400

[B16] Chalabi M. Verschoor Y. L. Tan P. B. Balduzzi S. Van Lent A. U. Grootscholten C. . (2024). Neoadjuvant immunotherapy in locally advanced mismatch repair-deficient colon cancer. N. Engl. J. Med. 390, 1949–1958. doi: 10.1056/nejmoa2400634 38838311

[B17] Chen D. Jin D. Huang S. Wu J. Xu M. Liu T. . (2020). Clostridium butyricum, a butyrate-producing probiotic, inhibits intestinal tumor development through modulating Wnt signaling and gut microbiota. Cancer Lett. 469, 456–467. doi: 10.1016/j.canlet.2019.11.019 31734354

[B18] Chen D. S. Mellman I. (2017). Elements of cancer immunity and the cancer-immune set point. Nature 541, 321–330. doi: 10.1038/nature21349 28102259

[B19] Cheng S. Han Z. Dai D. Li F. Zhang X. Lu M. . (2024). Multi-omics of the gut microbial ecosystem in patients with microsatellite-instability-high gastrointestinal cancer resistant to immunotherapy. Cell Rep. Med. 5, 101355. doi: 10.1016/j.xcrm.2023.101355 38194971 PMC10829783

[B20] Chuah L. O. Foo H. L. Loh T. C. Mohammed Alitheen N. B. Yeap S. K. Abdul Mutalib N. E. . (2019). Postbiotic metabolites produced by Lactobacillus plantarum strains exert selective cytotoxicity effects on cancer cells. BMC Complement Altern. Med. 19, 114. doi: 10.1186/s12906-019-2528-2 31159791 PMC6547513

[B21] Chung L. Orberg E. T. Geis A. L. Chan J. L. Fu K. DeStefano Shields C. E. . (2018). Bacteroides fragilis toxin coordinates a pro-carcinogenic inflammatory cascade via targeting of colonic epithelial cells. Cell Host Microbe 23, 203–214.e5. doi: 10.2139/ssrn.3155839 29398651 PMC5954996

[B22] ClinicalTrials.gov (2021). Fecal Microbiota Transplant and Re-Introduction of Anti-PD-1 Therapy (Pembrolizumab or Nivolumab) for the Treatment of Metastatic Colorectal Cancer in Anti-PD-1 non-Responders (Bethesda (MD: National Library of Medicine (US). Available online at: https://clinicaltrials.gov/study/NCT04729322 (Accessed June 10, 2026).

[B23] Cong J. Liu P. Han Z. Ying W. Li C. Yang Y. . (2024). Bile acids modified by the intestinal microbiota promote colorectal cancer growth by suppressing CD8+ T-cell effector functions. Immunity 57, 876–889.e11. doi: 10.1016/j.immuni.2024.02.014 38479384

[B24] Crespin A. Le Bescop C. de Gunzburg J. Vitry F. Zalcman G. Cervesi J. . (2023). Impact of antibiotic use on clinical outcomes of cancer patients treated with immune checkpoint inhibitors: a systematic review and meta-analysis. Front. Oncol. 13, 1075593. doi: 10.3389/fonc.2023.1075593 36937417 PMC10019357

[B25] Davar D. Dzutsev A. K. McCulloch J. A. Rodrigues R. R. Chauvin J. M. Morrison R. M. . (2021). Fecal microbiota transplant overcomes resistance to anti-PD-1 therapy in melanoma patients. Science 371, 595–602. doi: 10.1126/science.abf3363 33542131 PMC8097968

[B26] Derosa L. Routy B. Fidelle M. Iebba V. Alla L. Pasolli E. . (2020). Gut bacteria composition drives primary resistance to cancer immunotherapy in renal cell carcinoma patients. Eur. Urol. 78, 195–206. doi: 10.1016/j.eururo.2020.04.044 32376136

[B27] Dikeocha I. J. Al-Kabsi A. M. Chiu H. T. Alshawsh M. A. (2022). Faecalibacterium prausnitzii ameliorates colorectal tumorigenesis and suppresses proliferation of HCT116 colorectal cancer cells. Biomedicines 10, 1128. doi: 10.3390/biomedicines10051128 35625865 PMC9138996

[B28] Elkrief A. Pidgeon R. Maleki Vareki S. Messaoudene M. Castagner B. Routy B. . (2025). The gut microbiome as a target in cancer immunotherapy: opportunities and challenges for drug development. Nat. Rev. Drug Discov. 24, 685–704. doi: 10.1038/s41573-025-01211-7 40457025

[B29] Fidelle M. Rauber C. Alves Costa Silva C. Tian A. L. Lahmar I. de La Varende A. M. . (2023). A microbiota-modulated checkpoint directs immunosuppressive intestinal T cells into cancers. Science 380, eabo2296. doi: 10.1126/science.abo2296 37289890

[B30] Finotello F. Trajanoski Z. (2018). Quantifying tumor-infiltrating immune cells from transcriptomics data. Cancer Immunol. Immunother. 67, 1031–1040. doi: 10.1007/s00262-018-2150-z 29541787 PMC6006237

[B31] Gabrilovich D. I. (2017). Myeloid-derived suppressor cells. Nat. Rev. Immunol. 17, 711–726. doi: 10.1158/2326-6066.cir-16-0297 28052991 PMC5426480

[B32] Ghali E. N. H. K. Pranav Chauhan S. C. Yallapu M. M. (2024). Inulin-based formulations as an emerging therapeutic strategy for cancer: A comprehensive review. Int. J. Biol. Macromol. 259, 129216. doi: 10.1016/j.ijbiomac.2024.129216 38185294 PMC10922702

[B33] Gopalakrishnan V. Spencer C. N. Nezi L. Reuben A. Andrews M. C. Karpinets T. V. . (2018). Gut microbiome modulates response to anti-PD-1 immunotherapy in melanoma patients. Science 359, 97–103. doi: 10.1126/science.aan4236 29097493 PMC5827966

[B34] He Y. Fu L. Li Y. Wang W. Gong M. Zhang J. . (2021). Gut microbial metabolites facilitate anticancer therapy efficacy by modulating cytotoxic CD8+ T cell immunity. Cell Metab. 33, 988–1000.e7. doi: 10.1016/j.cmet.2021.03.002 33761313

[B35] Iliev I. D. Blander J. M. Collins N. Guo C. J. Longman R. S. Sonnenberg G. F. . (2025). Microbiota-mediated mechanisms of mucosal immunity across the lifespan. Nat. Immunol. 26, 1645–1659. doi: 10.1038/s41590-025-02281-w 40957908 PMC12700289

[B36] Jin Y. Dong H. Xia L. Yang Y. Zhu Y. Shen Y. . (2019). The diversity of gut microbiome is associated with favorable responses to anti-programmed death 1 immunotherapy in Chinese patients with NSCLC. J. Thorac. Oncol. 14, 1378–1389. doi: 10.1016/j.jtho.2019.04.007 31026576

[B37] Jin M. Wu J. Shi L. Zhou B. Shang F. Chang X. . (2022). Gut microbiota distinct between colorectal cancers with deficient and proficient mismatch repair: A study of 230 CRC patients. Front. Microbiol. 13, 993285. doi: 10.3389/fmicb.2022.993285 36312959 PMC9607965

[B38] Kalbasi A. Ribas A. (2020). Tumour-intrinsic resistance to immune checkpoint blockade. Nat. Rev. Immunol. 20, 25–39. doi: 10.1038/s41577-019-0218-4 31570880 PMC8499690

[B39] Knight R. Vrbanac A. Taylor B. C. Aksenov A. Callewaert C. Debelius J. . (2018). Best practices for analysing microbiomes. Nat. Rev. Microbiol. 16, 410–422. doi: 10.1038/s41579-018-0029-9 29795328

[B40] Komiya T. Huang C. H. (2018). Updates in the clinical development of epacadostat and other indoleamine 2,3-dioxygenase 1 inhibitors (IDO1) for human cancers. Front. Oncol. 8, 423. doi: 10.3389/fonc.2018.00423 30338242 PMC6180183

[B41] Kostic A. D. Chun E. Robertson L. Glickman J. N. Gallini C. A. Michaud M. . (2013). Fusobacterium nucleatum potentiates intestinal tumorigenesis and modulates the tumor-immune microenvironment. Cell Host Microbe 14, 207–215. doi: 10.1016/j.chom.2013.07.007 23954159 PMC3772512

[B42] Kudra A. Kaźmierczak-Siedlecka K. Sobocki B. K. Muszyński D. Połom J. Carbone L. . (2023). Postbiotics in oncology: science or science fiction? Front. Microbiol. 14, 1182547. doi: 10.3389/fmicb.2023.1182547 37608943 PMC10440707

[B43] Kumar R. Herold J. L. SChady D. Davis J. Kopetz S. Martinez-Moczygemba M. . (2017). Streptococcus gallolyticus subsp. gallolyticus promotes colorectal tumor development. PloS Pathog. 13, e1006440. doi: 10.1371/journal.ppat.1006440 28704539 PMC5509344

[B44] Le D. T. Durham J. N. Smith K. N. Wang H. Bartlett B. R. Aulakh L. K. . (2017). Mismatch repair deficiency predicts response of solid tumors to PD-1 blockade. Science 357, 409–413. doi: 10.1126/science.aan6733 28596308 PMC5576142

[B45] Limeta A. Ji B. Levin M. Gatto F. Nielsen J. (2020). Meta-analysis of the gut microbiota in predicting response to cancer immunotherapy in metastatic melanoma. JCI Insight 5, e140940. doi: 10.1172/jci.insight.140940 33268597 PMC7714408

[B46] Lin B. S. Yin M. Y. Xie S. A. Li P. Li X. (2026). Pleiotropic immunoregulation by bile acids in pathophysiology. Front. Immunol. 17, 1719092. doi: 10.3389/fimmu.2026.1719092 41727485 PMC12916643

[B47] Llosa N. J. Cruise M. Tam A. Wicks E. C. Hechenbleikner E. M. Taube J. M. . (2015). The vigorous immune microenvironment of microsatellite instable colon cancer is balanced by multiple counter-inhibitory checkpoints. Cancer Discov. 5, 43–51. doi: 10.1158/2159-8290.cd-14-0863 25358689 PMC4293246

[B48] Long X. Wong C. C. Tong L. Chu E. S. H. Ho Szeto C. Go M. Y. Y. . (2019). Peptostreptococcus anaerobius promotes colorectal carcinogenesis and modulates tumour immunity. Nat. Microbiol. 4, 2319–2330. doi: 10.1038/s41564-019-0541-3 31501538

[B49] Lu S. Xu J. Zhao Z. Guo Y. Zhang H. Jurutka P. W. . (2023). Dietary *Lactobacillus rhamnosus* GG extracellular vesicles enhance antiprogrammed cell death 1 (anti-PD-1) immunotherapy efficacy against colorectal cancer. Food Funct. 14, 10314–10328. doi: 10.18174/681298 37916395

[B50] Luu M. Riester Z. Baldrich A. Reichardt N. Yuille S. Busetti A. . (2021). Microbial short-chain fatty acids modulate CD8+ T cell responses and improve adoptive immunotherapy for cancer. Nat. Commun. 12, 4077. doi: 10.1038/s41467-021-24331-1 34210970 PMC8249424

[B51] Ma C. Han M. Heinrich B. Fu Q. Zhang Q. Sandhu M. . (2018). Gut microbiome-mediated bile acid metabolism regulates liver cancer via NKT cells. Science 360, eaan5931. doi: 10.1126/science.aan5931 29798856 PMC6407885

[B52] Mager L. F. Burkhard R. Pett N. Cooke N. C. A. Brown K. Ramay H. . (2020). Microbiome-derived inosine modulates response to checkpoint inhibitor immunotherapy. Science 369, 1481–1489. doi: 10.1126/science.abc3421 32792462

[B53] Marx V. (2021). Method of the year: spatially resolved transcriptomics. Nat. Methods 18, 9–14. doi: 10.1038/s41592-020-01033-y 33408395

[B54] Matson V. Fessler J. Bao R. Chongsuwat T. Zha Y. Alegre M. L. . (2018). The commensal microbiome is associated with anti-PD-1 efficacy in metastatic melanoma patients. Science 359, 104–108. doi: 10.1126/science.aao3290 29302014 PMC6707353

[B55] Murphy C. C. Zaki T. A. (2024). Changing epidemiology of colorectal cancer—birth cohort effects and emerging risk factors. Nat. Rev. Gastroenterol. Hepatol. 21, 25–34. doi: 10.1038/s41575-023-00841-9 37723270

[B56] Nicholson J. K. Holmes E. Kinross J. Burcelin R. Gibson G. Jia W. . (2012). Host-gut microbiota metabolic interactions. Science 336, 1262–1267. doi: 10.1126/science.1223813 22674330

[B57] Novielli P. Baldi S. Romano D. Magarelli M. Diacono D. Di Bitonto P. . (2025). Personalized colorectal cancer risk assessment through explainable AI and gut microbiome profiling. Gut Microbes 17, 2543124. doi: 10.1080/19490976.2025.2543124 40760681 PMC12326576

[B58] Odenwald M. A. Turner J. R. (2017). The intestinal epithelial barrier: a therapeutic target? Nat. Rev. Gastroenterol. Hepatol. 14, 9–21. doi: 10.1038/nrgastro.2016.169 27848962 PMC5554468

[B59] Overman M. J. McDermott R. Leach J. L. Lonardi S. Lenz H. J. Morse M. A. . (2017). Nivolumab in patients with metastatic DNA mismatch repair-deficient or microsatellite instability-high colorectal cancer (CheckMate 142). Lancet Oncol. 18, 1182–1191. doi: 10.1016/s1470-2045(17)30422-9 28734759 PMC6207072

[B60] Pallotta M. T. Rossini S. Suvieri C. Coletti A. Orabona C. Macchiarulo A. . (2022). Indoleamine 2,3-dioxygenase 1 (IDO1): an up-to-date overview of an eclectic immunoregulatory enzyme. FEBS J. 289, 6099–6118. doi: 10.1111/febs.16086 34145969 PMC9786828

[B61] Papalexi E. Satija R. (2018). Single-cell RNA sequencing to explore immune cell heterogeneity. Nat. Rev. Immunol. 18, 35–45. doi: 10.1038/nri.2017.76 28787399

[B62] Pleguezuelos-Manzano C. Puschhof J. Rosendahl Huber A. van Hoeck A. Wood H. M. Nomburg J. . (2020). Mutational signature in colorectal cancer caused by genotoxic pks+ Escherichia coli. Nature 580, 269–273. doi: 10.1038/s41586-020-2080-8 32106218 PMC8142898

[B63] Porcari S. Mullish B. H. Asnicar F. Ng S. C. Zhao L. Hansen R. . (2025). International consensus statement on microbiome testing in clinical practice. Lancet Gastroenterol. Hepatol. 10, 154–167. doi: 10.1016/s2468-1253(24)00311-x 39647502 PMC12343204

[B64] Roager H. M. Licht T. R. (2018). Microbial tryptophan catabolites in health and disease. Nat. Commun. 9, 3294. doi: 10.1038/s41467-018-05470-4 30120222 PMC6098093

[B65] Routy B. Le Chatelier E. Derosa L. Duong C. P. M. Alou M. T. Daillère R. . (2018). Gut microbiome influences efficacy of PD-1-based immunotherapy against epithelial tumors. Science 359, 91–97. doi: 10.1126/science.aan3706 29097494

[B66] Salminen S. Collado M. C. Endo A. Hill C. Lebeer S. Quigley E. M. M. . (2021). The International Scientific Association of Probiotics and Prebiotics (ISAPP) consensus statement on the definition and scope of postbiotics. Nat. Rev. Gastroenterol. Hepatol. 18, 649–667. doi: 10.1038/s41575-021-00440-6 33948025 PMC8387231

[B67] Serpas Higbie V. Rogers J. Hwang H. Qiao W. Xiao L. Dasari A. . (2022). Antibiotic exposure does not impact immune checkpoint blockade response in MSI-H/dMMR metastatic colorectal cancer: A single-center experience. Oncologist 27, 952–957. doi: 10.1093/oncolo/oyac162 35946836 PMC9632313

[B68] Sharma P. Hu-Lieskovan S. Wargo J. A. Ribas A. (2017). Primary, adaptive, and acquired resistance to cancer immunotherapy. Cell. 168, 707–723. doi: 10.1016/j.cell.2017.01.017 28187290 PMC5391692

[B69] Sinicrope F. A. Ou F. S. Zemla T. Nixon A. B. Mody K. Levasseur A. . (2019). Randomized trial of standard chemotherapy alone or combined with atezolizumab as adjuvant therapy for patients with stage III colon cancer and deficient mismatch repair (ATOMIC, Alliance A021502). J. Clin. Oncol. 37, e15169. doi: 10.1200/jco.2019.37.15_suppl.e15169 42586871

[B70] Sivan A. Corrales L. Hubert N. Williams J. B. Aquino-Michaels K. Earley Z. M. . (2015). Commensal Bifidobacterium promotes antitumor immunity and facilitates anti-PD-L1 efficacy. Science 350, 1084–1089. doi: 10.1126/science.aac4255 26541606 PMC4873287

[B71] Soheilipour M. Noursina A. Nekookhoo M. Malekpour E. Mirzaei S. A. Shafieyoun M. H. . (2025). The pathobiont role of Akkermansia muciniphila in colorectal cancer: a systematic review. BMC Gastroenterol. 25, 702. doi: 10.1186/s12876-025-04293-0 41062951 PMC12505710

[B72] Somodi C. Dora D. Horváth M. Szegvari G. Lohinai Z. (2025). Gut microbiome changes and cancer immunotherapy outcomes associated with dietary interventions: a systematic review of preclinical and clinical evidence. J. Transl. Med. 23, 756. doi: 10.1186/s12967-025-06586-0 40629403 PMC12239337

[B73] Spencer C. N. McQuade J. L. Gopalakrishnan V. McCulloch J. A. Vétizou M. Cogdill A. P. . (2021). Dietary fiber and probiotics influence the gut microbiome and melanoma immunotherapy response. Science 374, 1632–1640. doi: 10.1126/science.aaz7015 34941392 PMC8970537

[B74] Starling N. Elez E. Strickler J. H. Benson A. B. Oki E. Mendez G. . (2026). A phase III study of perioperative dostarlimab in patients with dMMR/MSI-H resectable colon cancer: AZUR-2 study design. Future Oncol. 22, 137–145. doi: 10.1080/14796694.2025.2606910 41492912 PMC12802981

[B75] Stemmer A. Margalit O. Serpas V. Strauss G. Thomas J. Shah P. . (2024). Immunotherapy in mismatch repair-deficient metastatic colorectal cancer: outcome and novel predictive markers. Eur. J. Cancer 198, 113495. doi: 10.1016/j.annonc.2023.09.1818 38157568

[B76] Su X. Gao Y. Yang R. (2023). Gut microbiota-derived bile acid metabolites maintain the homeostasis of gut and systemic immunity. Front. Immunol. 14, 1127743. doi: 10.3390/cells11152296 37256134 PMC10225537

[B77] Sung H. Filho A. M. Laversanne M. Ferlay J. Siegel R. L. Soerjomataram I. . (2026). Global cancer statistics 2024: GLOBOCAN estimates of incidence and mortality worldwide for 34 cancers in 186 countries. CA Cancer J. Clin. doi: 10.3322/caac.70090 42417444 PMC13343830

[B78] Ternes D. Karta J. Tsenkova M. Wilmes P. Haan S. Letellier E. (2020). Microbiome in colorectal cancer: how to get from meta-omics to mechanism? Trends Microbiol. 28, 401–423. doi: 10.1016/j.tim.2020.05.013 32298617

[B79] Tofalo R. Cocchi S. Suzzi G. (2019). Polyamines and gut microbiota. Front. Nutr. 6, 16. doi: 10.3389/fnut.2019.00016 30859104 PMC6397830

[B80] Tran M. Huh J. R. Devlin A. S. (2025). The role of gut microbial metabolites in the T cell lifecycle. Nat. Immunol. 26, 1246–1257. doi: 10.1038/s41590-025-02227-2 40691327 PMC13124069

[B81] Tremblay J. Schreiber L. Greer C. W. (2022). High-resolution shotgun metagenomics: the more data, the better? Brief Bioinform. 23, bbac443. doi: 10.1093/bib/bbac443 36352504

[B82] Tukenmez U. Aktas B. Aslim B. Yavuz S. (2019). The relationship between the structural characteristics of lactobacilli-EPS and its ability to induce apoptosis in colon cancer cells *in vitro*. Sci. Rep. 9, 8268. doi: 10.1038/s41598-019-44753-8 31164685 PMC6547643

[B83] van der Leun A. M. Thommen D. S. Schumacher T. N. (2020). CD8^+^ T cell states in human cancer: insights from single-cell analysis. Nat. Rev. Cancer 20, 218–232. doi: 10.1016/b978-012078185-0/50439-x 32024970 PMC7115982

[B84] Vétizou M. Pitt J. M. Daillère R. Lepage P. Waldschmitt N. Flament C. . (2015). Anticancer immunotherapy by CTLA-4 blockade relies on the gut microbiota. Science 350, 1079–1084. doi: 10.1126/science.aad1329 26541610 PMC4721659

[B85] Veziant J. Bonnet M. Occean B. V. Dziri C. Pereira B. Slim K. (2022). Probiotics/Synbiotics to Reduce Infectious Complications after Colorectal Surgery: A Systematic Review and Meta-Analysis of Randomised Controlled Trials. Nutrients 14, 3066. doi: 10.3390/nu14153066 35893922 PMC9332115

[B86] Wang T. Gnanaprakasam J. N. R. Chen X. Kang S. Xu X. Sun H. . (2020). Inosine is an alternative carbon source for CD8^+^-T-cell function under glucose restriction. Nat. Metab. 2, 635–647. doi: 10.1038/s42255-020-0219-4 32694789 PMC7371628

[B87] Wong S. H. Yu J. (2019). Gut microbiota in colorectal cancer: mechanisms of action and clinical applications. Nat. Rev. Gastroenterol. Hepatol. 16, 690–704. doi: 10.1038/s41575-019-0209-8 31554963

[B88] Wu D. Zhu Y. (2021). Role of kynurenine in promoting the generation of exhausted CD8^+^ T cells in colorectal cancer. Am. J. Transl. Res. 13, 1535–1547. 33841677 PMC8014392

[B89] Xi J. Li Z. Liu Y. Niu W. Yu B. Li Z. . (2026). Dissecting immunosuppressive microenvironment in chemotherapy-resistant colorectal cancer through single-cell transcriptomic analysis. Transl. Oncol. 71, 102889. doi: 10.1016/j.tranon.2026.102889 42447566 PMC13377434

[B90] Xie M. Yuan K. Zhang Y. Zhang Y. Zhang R. Gao J. . (2025). Tumor-resident probiotic Clostridium butyricum improves aPD-1 efficacy in colorectal cancer models by inhibiting IL-6-mediated immunosuppression. Cancer Cell. 43, 1885–1901. doi: 10.1016/j.ccell.2025.07.012 40780216

[B91] Xie W. Zhong Y. S. Li X. J. Kang Y. K. Peng Q. Y. Ying H. Z. (2024). Postbiotics in colorectal cancer: intervention mechanisms and perspectives. Front. Microbiol. 15, 1360225. doi: 10.3389/fmicb.2024.1360225 38450163 PMC10914944

[B92] Yachida S. Mizutani S. Shiroma H. Shiba S. Nakajima T. Sakamoto T. . (2019). Metagenomic and metabolomic analyses reveal distinct stage-specific phenotypes of the gut microbiota in colorectal cancer. Nat. Med. 25, 968–976. doi: 10.1038/s41591-019-0458-7 31171880

[B93] Zeng J. Ji Y. Liang B. Zhang G. Chen D. Zhu M. . (2021). The effect of pro/synbiotics on postoperative infections in colorectal cancer patients: A systematic review and meta-analysis. Complement Ther. Clin. Pract. doi: 10.1016/j.ctcp.2021.101370 33894576

[B94] Zeng Y. Shi Q. Liu X. Tang H. Lu B. Zhou Q. . (2023). Dynamic gut microbiota changes in patients with advanced Malignancies experiencing secondary resistance to immune checkpoint inhibitors and immune-related adverse events. Front. Oncol. 13, 1144534. doi: 10.3389/fonc.2023.1144534 37114123 PMC10126279

[B95] Zhang X. Li L. Butcher J. Stintzi A. Figeys D. (2019). Advancing functional and translational microbiome research using meta-omics approaches. Microbiome 7, 154. doi: 10.1186/s40168-019-0767-6 31810497 PMC6898977

[B96] Zhang S. L. Mao Y. Q. Zhang Z. Y. Li Z. M. Kong C. Y. Chen H. L. . (2021). Pectin supplement significantly enhanced the anti-PD-1 efficacy in tumor-bearing mice humanized with gut microbiota from patients with colorectal cancer. Theranostics 11, 4155–4170. doi: 10.7150/thno.54476 33754054 PMC7977465

[B97] Zhao W. Lei J. Ke S. Chen Y. Xiao J. Tang Z. . (2023). Fecal microbiota transplantation plus tislelizumab and fruquintinib in refractory microsatellite stable metastatic colorectal cancer: an open-label, single-arm, phase II trial (RENMIN-215). EClinicalMedicine. 66, 102315. doi: 10.1016/j.eclinm.2023.102315 38024475 PMC10679864

[B98] Zhong B. Zhao Y. Gao L. Yang G. Gao Y. Li F. . (2024). Anticancer Effects of *Weizmannia coagulans* MZY531 Postbiotics in CT26 Colorectal Tumor-Bearing Mice by Regulating Apoptosis and Autophagy. Life. (Basel). 14, 1334. doi: 10.3389/fphar.2020.611087 39459634 PMC11509727

[B99] Zhu X. Hu M. Huang X. Li L. Lin X. Shao X. . (2025). Interplay between gut microbial communities and metabolites modulates pan-cancer immunotherapy responses. Cell Metab. 37, 806–823. doi: 10.1016/j.cmet.2024.12.013 39909032

[B100] Zhuo Q. Yu B. Zhou J. Zhang J. Zhang R. Xie J. . (2019). Lysates of Lactobacillus acidophilus combined with CTLA-4-blocking antibodies enhance antitumor immunity in a mouse colon cancer model. Sci. Rep. 9, 20128. doi: 10.1038/s41598-019-56661-y 31882868 PMC6934597

